# PR inhibition stimulates G6PD expression to enhance malignancy in luminal breast cancer

**DOI:** 10.1038/s41419-025-08365-7

**Published:** 2025-12-21

**Authors:** Jae Woong Jeong, Janghee Lee, Soong June Bae, Yoon Jin Cha, Sungsoon Fang, Hae-kyung Lee, Sung Gwe Ahn

**Affiliations:** 1 Institute for Immunology and Immunological Diseases, Yonsei University College of Medicine, Seoul, Republic of Korea; 2 Department of Medicine, Yonsei University College of Medicine, Seoul, Republic of Korea; 3 Department of Surgery, Ewha Womans University Mokdong Hospital, Ewha Womans University College of Medicine, Seoul, Republic of Korea; 4 Department of Surgery, Breast Cancer Center, Gangnam Severance Hospital, Yonsei University College of Medicine, Seoul, Republic of Korea; 5 Institute for Breast Cancer Precision Medicine, Yonsei University College of Medicine, Seoul, Republic of Korea; 6 Department of Pathology, Gangnam Severance Hospital, Yonsei University College of Medicine, Seoul, Republic of Korea; 7 Department of Biomedical Sciences, Gangnam Severance Hospital, Yonsei University College of Medicine, Seoul, Republic of Korea; 8 Severance Biomedical Science Institute, Gangnam Severance Hospital, Yonsei University College of Medicine, Seoul, Republic of Korea

**Keywords:** Breast cancer, Cell growth

## Abstract

Luminal breast cancer is the most prevalent and prognostic subtype of breast cancer. However, it has been reported that luminal breast cancer patients with lower progesterone receptor (PR) expression are associated with poor survival outcomes. Nevertheless, there is insufficient evidence linking PR expression to an aggressiveness of luminal breast cancer. Based on our previous studies showing an inverse correlation between PR and standardized uptake value (SUV) on [18 F] fluorodeoxyglucose positron emission tomography (FDG-PET), we aimed to identify a potential link between PR expression and glucose metabolism, particularly the pentose phosphate pathway (PPP). To investigate it, we performed a single cell RNA sequencing (scRNA-seq) analysis using published dataset. Interestingly, the analysis revealed that specific epithelial cells with both increased proliferation activity and decreased PR expression, which increased activity of the PPP and glucose-6-phosphate dehydrogenase (G6PD) expression. To verify these findings, we silenced PR expression in the luminal breast cancer cell lines, MCF7 and T47D, which led to accelerated proliferation and PPP activity with G6PD expression. We hypothesized that PR knockdown (KD) increases breast cancer aggressiveness by boosting glucose utilization with PPP activity. Importantly, treatment with G6PD inhibitor (G6PDi), a G6PDi reduced aggressiveness of PR KD cancer cells. These findings suggest that targeting G6PD could be a promising therapeutic strategy to suppress the aggressiveness of luminal breast cancer, using low PR expression as a biomarker.

## Introduction

Breast cancer, one of the most prevalent cancers worldwide, is clinically categorized into four subtypes. These subtypes include luminal-like A, luminal-like B, human epidermal growth factor receptor 2 (HER2)-positive and triple negative breast cancer, determined by immunohistochemical (IHC) intensity of hormone receptors and HER2 [1]. This IHC-based classification (such as luminal-like A, luminal-like B) is distinct from the PAM50 gene expression-based intrinsic subtypes (such as Luminal A, Luminal B), though they are often correlated [2]. Our study investigates “luminal breast cancer” as a broad category that includes subtypes defined by both methods. To ensure methodological precision throughout this manuscript, we distinguish between the “luminal” intrinsic subtypes (PAM50) and the “luminal-like” clinical subtypes (IHC). The luminal-like subtypes (A and B) are characterized by hormone receptor-positive and HER2-negative status, making them the most common subtype with a generally better prognosis compared to others [3–5]. However, concerns such as late recurrence still exist for these patients, highlighting the ongoing need for novel therapeutic strategies [6].

The progesterone receptor (PR) is a well-established prognostic factor in estrogen receptor (ER)-positive/HER2-negative breast cancer. Previous clinical studies have reported that tumors without PR expression increase recurrence and mortality rates compared to tumors with PR expression [7, 8]. Furthermore, the PR gene is included in the 21-multigene assay [9, 10], a standard test for predicting the benefit of chemotherapy in ER-positive/HER2-negative breast cancer, and it has been demonstrated that there is inverse correlation between PR expression and the recurrence score (RS) of 21-multigene assay (Oncotype DX^®^) [11]. However, the mechanism of PR as a prognostic factor has not been clearly elucidated.

Our previous findings revealed an inverse correlation between PR expression and standardized uptake value (SUV) on [^18^F] fluorodeoxyglucose positron emission tomography (FDG-PET) scans [11, 12]. This suggests a potential link between low PR levels, increased glucose uptake, and aggressiveness of breast cancer [13]. Elevated glucose uptake, as indicated by high SUV, implicates reliance of cancer cells on glycolysis for energy production [14].

The pentose phosphate pathway (PPP), the parallel pathway to glycolysis, is crucial for managing oxidative stress through NADPH generation [15]. The PPP is activated in human cancer tissues and is known to enhance the malignant potential of cancer cells, including cell proliferation, tumor invasion, and therapy resistance [16]. Importantly, high glucose-6-phosphate dehydrogenase (G6PD), the key enzyme of PPP, correlates with poor prognosis in breast cancer patients [17]. Given the association between low PR, increased glucose utilization, and aggressiveness of breast cancer, we hypothesized that low PR expression in luminal breast cancer might activate the PPP through G6PD.

In this study, we investigated the relation between PR and breast cancer malignancy and determined which signaling pathways influence the aggressiveness in cancer cells. Interestingly, our analysis identified a population of aggressive luminal epithelial cells characterized by low PR expression and an enrichment of the PPP. To verify this finding in vitro, we silenced PR on luminal breast cancer cell lines and performed bulk mRNA sequencing (bulk RNA-seq), finding up-regulation of G6PD expression. Furthermore, we elucidated the transcriptional mechanistic link by which PR loss leads to G6PD upregulation, potentially mediated by redox-sensitive transcriptional control such as NRF2 [18]. Critically, treatment with a selective G6PD inhibitor (G6PDi) [19] reversed this aggressive phenotype, significantly decreasing the growth rate of PR KD cells. These findings suggest that G6PD might be a novel target in luminal breast cancer with low PR expression as a biomarker.

## Results

### scRNA-seq analysis sorting luminal epithelial cells in luminal breast cancer

**Fig. 1:**
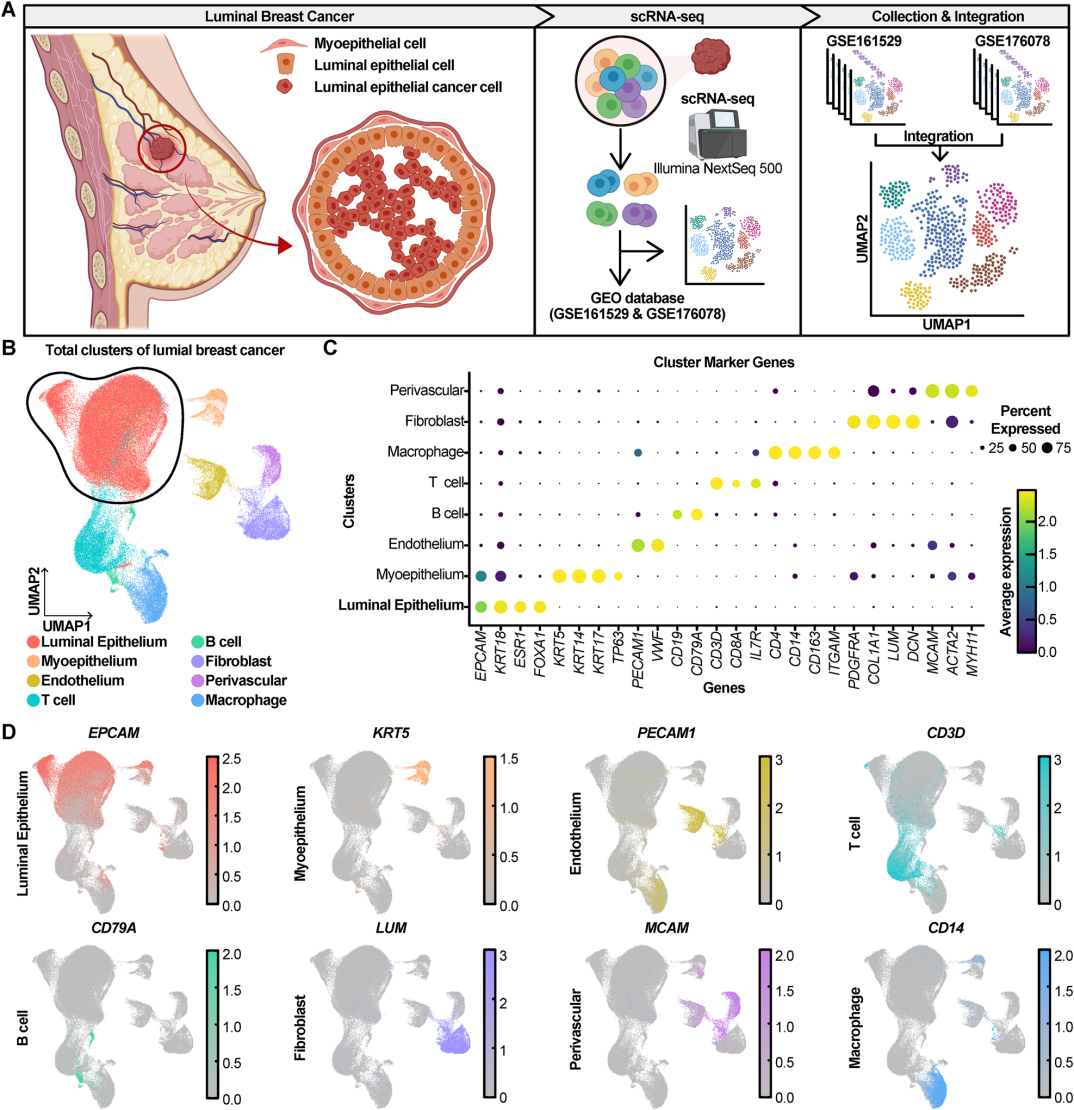
scRNA-seq analysis categorizing eight cell types in luminal breast cancer patient data. **A** Schematic diagram illustrating the cellular organization of luminal breast cancer and the workflow of scRNA-seq analysis. **B** UMAP plot presenting identified cell types. **C**, **D** Dot plot and feature plots showing marker genes for each cell type.

The luminal epithelial cells and myoepithelial cells are components of the breast duct system [20]. It has been suggested that breast cancers originate from luminal progenitors [21–25]. In contrast, the myoepithelial cells serve as a barrier to the invasion and dissemination of luminal epithelial cancer cells [26]. To investigate the relation of PR, PPP, and aggressiveness in luminal breast cancer, we performed scRNA-seq analysis using published data from the Gene Expression Omnibus (GEO) database (Fig. 1A).

First, we collected 33 luminal-like breast cancer patient matrices of scRNA-seq data based on the 10x Genomics platform from GSE161529 [27] and GSE176078 [28]. These matrices were processed using Seurat package in R. Through the process of quality control and integration, approximately 100,000 cells were used in analysis.

Then, to visualize these cells on two-dimensions using uniform manifold approximation and projection (UMAP), principal component analysis (PCA) was performed (Fig. 1B). It presented that about 100,000 cells were divided into eight clusters and these clusters had distinctive identities of characteristic lineage cell with marker genes. These include *EPCAM*, *KRT18*, *ESR1*, *FOXA1* for luminal epithelial cell, *KRT5*, *KRT14*, *KRT17*, *TP63* for myoepithelial cell, *PEACAM1*, *VWF* for endothelium, *CD3D*, *CD8A*, *IL7A* for T cell, *CD19*, *CD79A* for B cell, *PDGFRA*, *COL1A1*, *LUM*, *DCN* for fibroblasts, *MCAM*, *ACTA2*, *MYH11* for perivascular cell, and *CD4*, *CD14*, *CD163*, *ITGAM* for macrophage, as observed across different clusters (Fig. 1C and D). Among eight cell types, the luminal epithelial cell cluster, which is the dominant cancer cell in luminal breast cancer [21–25], was subsetted (Fig. S1A).

### Luminal epithelial cells divided by cell cycle rate and PR expression

**Fig. 2:**
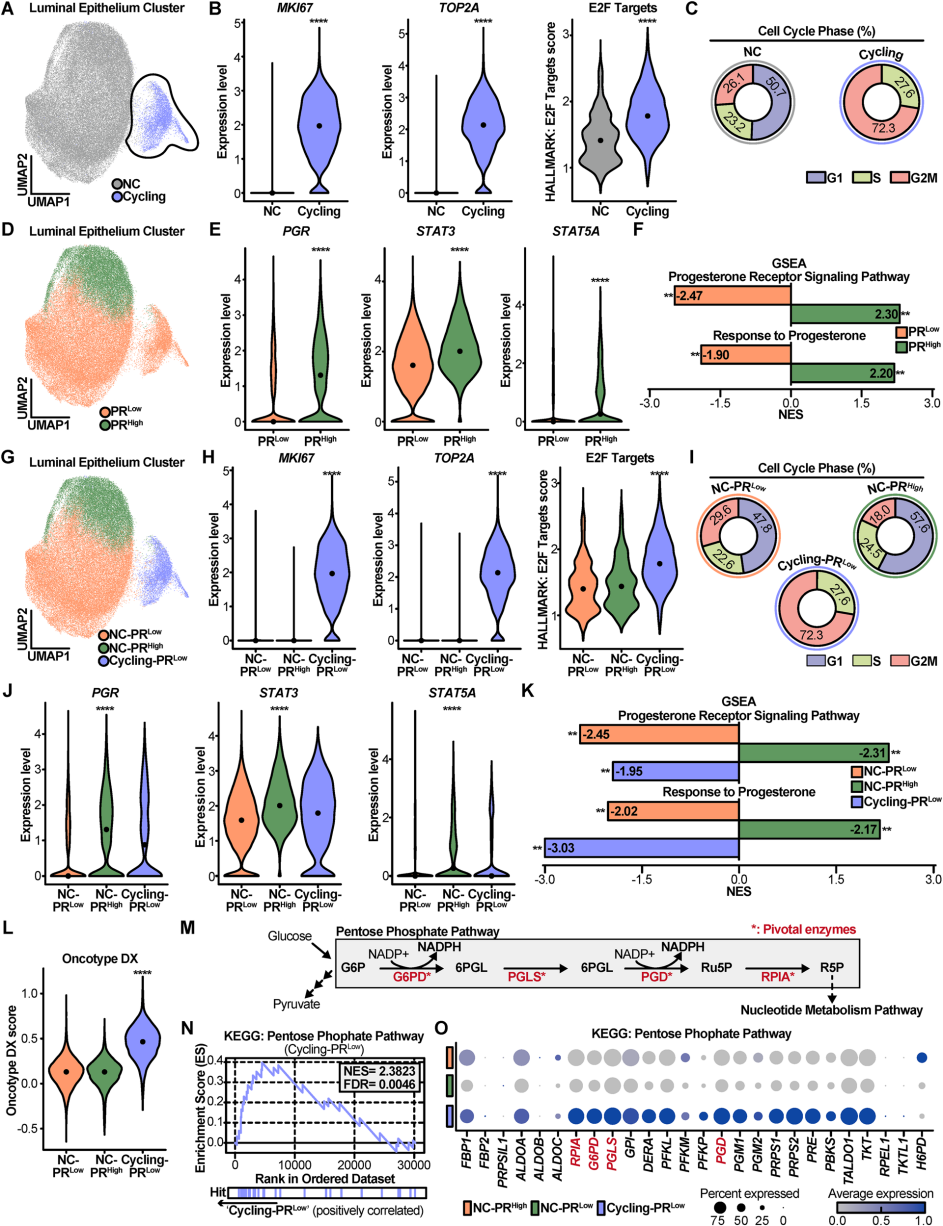
A proliferative low-PR subpopulation of luminal epithelial cells is characterized by heightened PPP activity. **A** UMAP plot displaying Non-cycling (NC) and Cycling groups. **B** Violin plots comparing the expression of proliferation markers (*MKI67*, *TOP2A*) and the E2F targets pathway score between NC and Cycling groups. **C** Donut charts illustrating the proportion of cells in each cell cycle phase for NC and Cycling groups. **D** UMAP plot showing PR ^Low^ and PR ^High^ groups. **E** Violin plots comparing the expression levels of *PGR* and its downstream targets (*STAT3*, *STAT5A*) between PR ^Low^ and PR ^High^ groups. **F** Bar plot exhibiting NES of progesterone related pathways between PR ^Low^ and PR ^High^ groups. **G** UMAP plot displaying the three subgroups: NC-PR ^High^, NC-PR ^Low^, and Cycling-PR ^Low^. **H**, **I** Violin plot and corresponding donut charts confirming that the Cycling-PR ^Low^ group has the highest expression of proliferation markers and the greatest proportion of cells in the S and G2/M phases. **J**, **K** Violin plots and bar plots showing diminished PGR expression and suppressed progesterone signaling in the Cycling-PR ^Low^ group. **L** Violin plot showing that the Oncotype DX (RS) is significantly elevated in the Cycling-PR ^Low^ group. **M** Schematic diagram of the PPP highlighting key enzymes. **N** GSEA enrichment plot showing significant activation of the PPP in the Cycling-PR ^Low^ group. **O** Dot plot showing the expression of genes within the PPP gene set across the three subgroups. For all violin plots, the line represents the median value. Statistical significance was determined using a Wilcoxon rank-sum test, and adjusted *p*-values are shown. * *p* < 0.05, ** *p* < 0.01, *** *p* < 0.001, **** *p* < 0.0001.

The most interesting aspect of the luminal epithelial cell subcluster was its positioning within a right-sided island (black dotted line), as illustrated in the UMAP (Fig. 2A). This island part showed significantly increased proliferation marker genes, *MKI67* [29] and *TOP2A* [30] (Fig. 2B). In addition, the island part exhibited an elevated ‘E2F Targets’ score based on Hallmark database [31] using gene set enrichment analysis (GSEA) [32] (Fig. 2B). The serial data indicated that the island cells were proliferative, leading to these island cells being designated as Cycling group and the rest of cells as non-cycling (NC) group. To confirm the Cycling group was undergoing proliferation, cell cycle phase analysis was conducted. It revealed that the Cycling group exhibited a much higher percentage of G2M (72.3%) phases than NC (26.1%) (Fig. 2C). Therefore, these proliferative cells were regarded as representative of malignant luminal epithelial cells.

Interestingly, the luminal epithelial cell cluster also could be separated by *PGR* expression (Fig. 2D), leading to two distinct groups, PR ^Low^ group and PR ^High^ group. As expected, the PR ^Low^ group exhibited statistically lower expression levels of *PGR* than the PR ^High^ group. And direct target genes of PR [33], *STAT3* and *STAT5A*, were significantly diminished in the PR ^Low^ group (Fig. 2E). In addition, PR ^Low^ group carried with negative normalized enrichment score (NES) of Progesterone Receptor Signaling Pathway (NES = −2.47) and Response to Progesterone (NES = −1.90) based on KEGG database using GSEA [34] (Fig. 2F). This data demonstrated that PR ^Low^ group had down-regulated activity of PR related signaling. This dual classification based on proliferation and PR expression provided a robust framework to investigate tumor aggressiveness.

### Proliferative luminal epithelial cells with PR-low exhibit upregulated PPP activity and G6PD expression

An intriguing phenomenon was found that all cells in the Cycling group belonged to the PR ^Low^ group (Fig. 2A and D). To further analyze this phenomenon, three categorized cell clusters were designated as NC-PR ^Low^, NC-PR ^High^ and Cycling-PR ^Low^ groups (Figs. S1B, 2G). Cycling-PR ^Low^ cluster maintained their proliferative characteristics (Fig. 2H and I), while exhibiting low expression of *PGR*, *STAT3* and *STAT5A*, as well as negative NES of progesterone-related signaling compared to NC-PR ^High^ (Fig. 2J and K). These findings indicate that the Cycling-PR ^Low^ cells represent an aggressive phenotype with diminished PR activity.

The most interesting observation was that RS of 21-multigene assay [9, 10], which predicts the recurrence and aggressiveness of breast cancer, was significantly elevated in the Cycling-PR ^Low^ cluster (Fig. 2L). In contrast, the difference of RS between the NC-PR ^Low^ and NC-PR ^High^ was not significant (Fig. 2L).

To investigate the lack of evidence for an inverse relation of PR and PPP, the subclusters were analyzed with a focus on PPP. PPP functions through pivotal enzymes, including G6PD and PGD, which form NADPH, as well as PGLS and RPIA, which also directly interact with metabolites [15] (Fig. 2M). To analyze these pivotal enzymes, the PPP gene set from the KEGG pathway was used. The Cycling-PR ^Low^ cluster exhibited significantly upregulated PPP activity, with positive NES of 2.3823 and false discovery rate (FDR) of 0.0046 (Fig. 2N). Furthermore, the expression of genes in the PPP gene set was elevated in the Cycling-PR ^Low^ cluster (Fig. 2O). Among these, the pivotal enzymes were significantly upregulated in Cycling-PR ^Low^ cluster (Fig. S1C). Taken together, our scRNA-seq analysis demonstrates that proliferative PR-low luminal epithelial cells exhibit heightened PPP activity and G6PD expression.

To further validate these findings from a genomic perspective, copy number variation (CNV) score analysis was performed. The CopyKAT package [35] in R was utilized to classify the epithelial cells into aneuploid, diploid, and undefined cells (Fig. S2A) and Aneuploid cells, which exhibited an abnormal number of chromosomes with high CNV score, were subsetted (Fig. S2B). These aneuploid cells were then divided into NC-PR ^Low^, NC-PR ^High^, and Cycling-PR ^Low^ groups for further analysis. This aneuploid subset recapitulated our previous findings, showing similar patterns of PR and G6PD expression, Oncotype DX (RS), and PPP activity across the NC-PR ^Low^, NC-PR ^High^, and Cycling-PR ^Low^ groups (Fig. S2C).

### Clinical implication of reverse association between PR and G6PD in luminal breast cancer

**Fig. 3:**
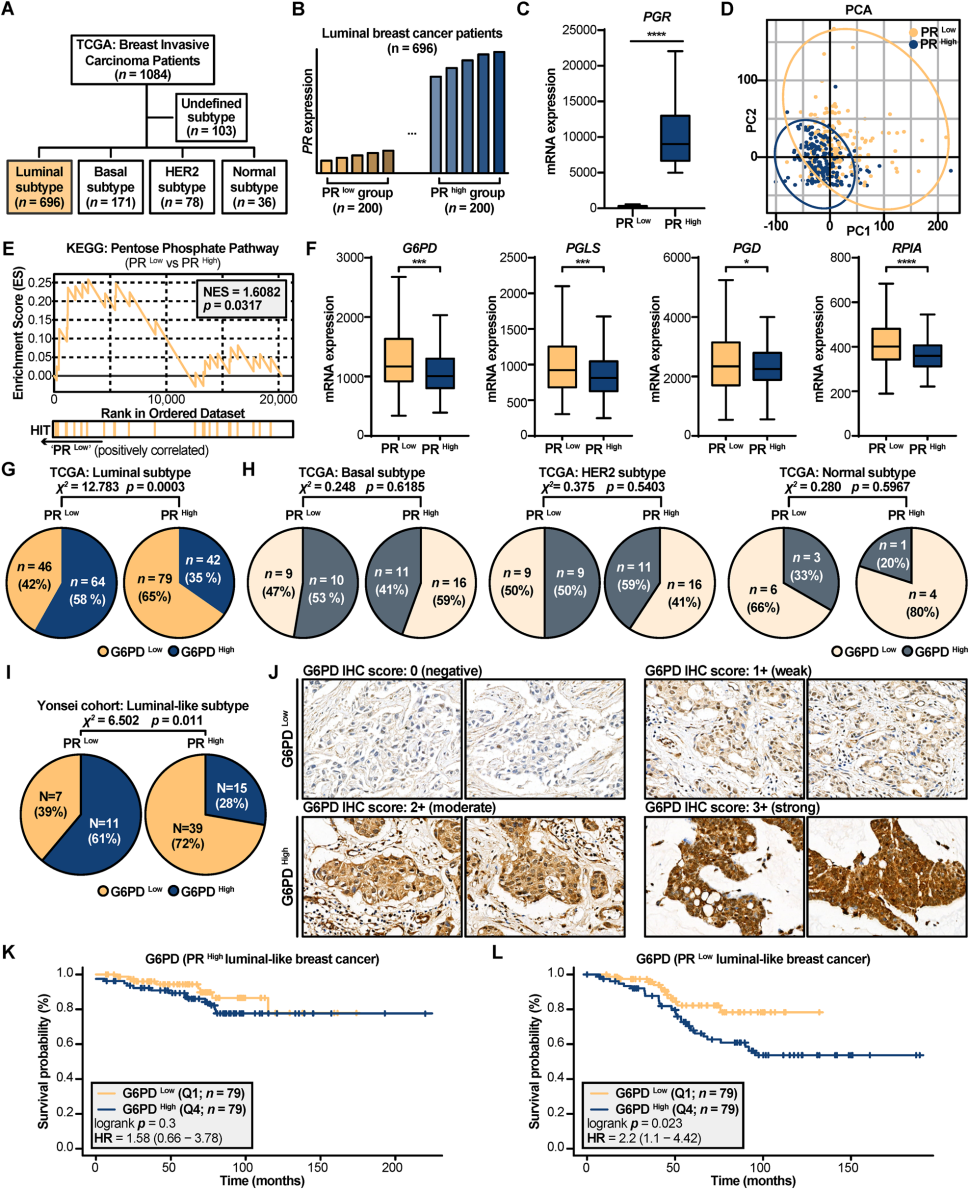
Low PR expression is clinically associated with elevated G6PD/PPP activity and poor prognosis in luminal breast cancer. **A** Schematic diagram illustrating the composition of the TCGA Breast Invasive Carcinoma cohort (*n* = 1084), highlighting the luminal subtype (*n* = 696) used for subsequent analyses. **B** Bar diagram illustrating the stratification of luminal patients into PR ^Low^ (*n* = 200) and PR ^High^ (*n* = 200) groups based on *PGR* expression. **C** Box plot confirming lower *PGR* expression in the PR ^Low^ group. **D** PCA plot of transcriptomic data showing distinct clustering between PR ^Low^ and PR ^High^ groups. **E** GSEA enrichment plot showing significant enrichment of the PPP in the PR ^Low^ group. **F** Box plots showing increased expression of key PPP enzymes (*G6PD*, *PGLS*, *PGD*, *RPIA*) in the PR ^Low^ group. **G**, **H** Pie charts showing the proportion of G6PD ^High^ and G6PD ^Low^ patients within the PR ^Low^ and PR ^High^ groups. A significant association is observed in the luminal/luminal-like subtype but not in other subtypes as determined by a Chi-square test. **I**, **J** Validation in an independent cohort from the Yonsei cohort (*n* = 72). **I** Pie chart showing a significant association between low PR status and high G6PD expression. **J** Representative IHC images showing protein expression of G6PD (magnification × 400) from the Yonsei cohort. **K**, **L** Kaplan–Meier survival curves for luminal breast cancer patients, stratified by G6PD expression. **K** No significant survival difference is observed in the PR ^High^ group. **L** In the PR ^Low^ group, high G6PD expression is significantly associated with poorer overall survival. For box plots, the line represents the mean value. Statistical significance was determined using an unpaired two-tailed t-test. For categorical data in pie charts, associations were assessed using the Chi-square test. * *p* < 0.05, ** *p* < 0.01, *** *p* < 0.001, **** *p* < 0.0001.

To study association between G6PD and PR expression in luminal breast cancer patients, data from The Cancer Genome Atlas Program (TCGA): Breast Invasive Carcinoma Patients [36] was used. A total of 1084 breast cancer patients were included in the study, of whom 696 were classified as having luminal subtypes. The other subtypes included basal-like (*n* = 171), HER2-enriched (*n* = 78), normal-like (*n* = 36), and undefined (*n* = 103) cases (Fig. 3A).

Then, the luminal subtype patients were divided into two groups, PR ^Low^ (*n* = 200) and PR ^High^ (*n* = 200), based on their *PGR* expression level (Fig. 3B). The group division was confirmed with a visualization of *PGR* expression (Fig. 3C). Interestingly, PCA revealed distinct transcriptomic profiles between the PR ^Low^ and PR ^High^ groups, despite the grouping being based solely on *PGR* expression (Fig. 3D). In addition, PR ^Low^ patients showed significantly upregulated PPP activity (NES = 1.6082 and *p* = 0.0317) (Fig. 3E). Furthermore, the expression levels of the pivotal enzymes of PPP, *G6PD*, *PGLS*, *PGD*, and *RPIA* (Fig. 2M), were all significantly increased in the PR ^Low^ group (Fig. 3F).

Upon closer inspection of the PCA plot (Fig. 3D), we noted that a substantial number of patients clustered near the origin of the principal component axes. This distribution suggested the presence of significant transcriptomic heterogeneity within both the PR ^Low^ and PR ^High^ groups, which could potentially dilute the core biological differences between them. To address it and to isolate the most distinct molecular phenotypes, we performed a refined sub-analysis.

Therefore, we created new subsets by selecting only the patients with the most divergent scores along the PC1 axis, representing the most transcriptionally divergent individuals in each cohort (Fig. S3A). This strategy proved highly effective at resolving the issue of heterogeneity. Hierarchical clustering (Euclidean distance, complete linkage) of these divergent subgroups demonstrated a clear and distinct segregation into two clusters (Fig. S3B). The enhanced separation was also evident in the differential expression analysis, where a volcano plot revealed a more pronounced transcriptomic divergence (Fig. S3C). Critically, the expression differences of key PPP-related genes became starker. The upregulation of *G6PD* and *PGLS* in the PR ^Low^ divergent group was highly significant (*p* = 2.63E-06 and *p* = 9.78E-05), and *PGD* and *RPIA* also showed a clear trend of increased expression (*p* = 0.0939 and *p* = 0.1170), contributing to a more pronounced overall activation pattern of the pathway (Fig. S3D). Reinforcing these observations, a subsequent GSEA confirmed that the PPP was now more significantly enriched in the PR ^Low^ divergent group (NES = 1.5407, FDR = 0.00446) (Fig. S3E). Taken together, these results demonstrate that by focusing the analysis on the most transcriptionally divergent patients, the confounding effects of intra-group heterogeneity are minimized, revealing a definitive and robust association between low PR status and the activation of the PPP.

To assess whether PR ^Low^ patients are more likely to exhibit high G6PD expressions across different breast cancer subtypes, they were stratified by *PGR* expression and further categorized into G6PD ^Low^ and G6PD ^High^ groups. A Chi-square test revealed a significant association in the luminal subtype (*χ*² = 12.783, *p* = 0.0003), indicating that PR ^Low^ patients had a higher proportion of G6PD ^High^ expression cases (Fig. 3G). In contrast, no significant association was observed in the other subtypes (Fig. 3H). When comparing *G6PD*, *PGLS*, *PGD*, and *RPIA* expression between PR ^Low^ and PR ^High^ groups within the basal-like, HER2-enriched, and normal-like subtypes, no notable differences were found—except for *RPIA* in the basal-like subtype (Fig. S4A–C). KEGG analysis of clinical data suggested that luminal subtype patients with low PR expression were characterized by enriched PPP and increased G6PD expression, whereas such patterns were not observed in other subtypes.

Here, we presented those 72 patients’ data in Yonsei cohort diagnosed with luminal-like breast cancer with clinical PR status and intensity score of G6PD using immunohistochemistry (IHC). The 72 patients were divided into PR ^Low^ (*n* = 18), PR ^High^ (*n* = 54) based on PR status, and they were split into G6PD ^Low^ (*n* = 46), G6PD ^High^ (*n* = 26) based on G6PD score (Fig. 3I and J, Fig. S5A–D). Among the 72 patients, a Chi-square test demonstrated a significant association between PR status and G6PD expression (*χ*² = 6.502, *p* = 0.011), indicating that high G6PD expression was more frequent in PR ^Low^ patients, while low G6PD expression predominated in PR ^High^ patients (Fig. 3I).

It is noteworthy, however, that a substantial subset of these PR ^Low^ patients remained in the G6PD ^Low^ category (Fig. 3G and I), highlighting a degree of intra-group heterogeneity. This suggests the link between PR loss and G6PD expression may be influenced by additional factors.

To ascertain the survival probabilities of luminal-like breast cancer patients in relation to PR and G6PD expression, we employed the Kaplan–Meier Plotter web tool, which utilizes large-scale publicly available datasets [37]. The patients were divided into PR ^Low^ (quartiles; Q1; *n* = 272) and PR ^High^ (Q4; *n* = 269) groups based on *PGR* expression levels. The PR ^High^ group (Q4) exhibited significantly better survival probability compared to the PR ^Low^ group, with a hazard ratio of 0.45 (*p* = 0.0002) (Fig. S6A). Conversely, patients in the G6PD ^High^ group (Q4; *n* = 269) showed significantly poorer survival probability than those in the G6PD ^Low^ group (Q1; *n* = 270), with a hazard ratio of 1.93 (*p* = 0.0029) (Fig. S6B). Importantly, among PR ^High^ patients, no statistically significant difference in survival probability was observed between the G6PD ^Low^ (Q1; *n* = 79) and G6PD ^High^ (Q4; *n* = 79) subgroups (*p* = 0.3) (Fig. 3K). In contrast, among PR ^Low^ patients, those with high G6PD expression (Q4; *n* = 79) exhibited significantly reduced survival probability compared to those with low G6PD expression (Q1; *n* = 79), with a hazard ratio of 2.2 (*p* = 0.023) (Fig. 3L). These results suggest that the impact of G6PD expression on survival may be greater in patients with low PR expression.

Collectively, these analyses of TCGA data, an independent clinical cohort, and survival outcomes establish a consistent clinical association between low PR expression, elevated PPP/G6PD activity, and poorer prognosis in patients with luminal breast cancer.

### Aggressiveness induction through PR KD in luminal breast cancer cell lines

**Fig. 4:**
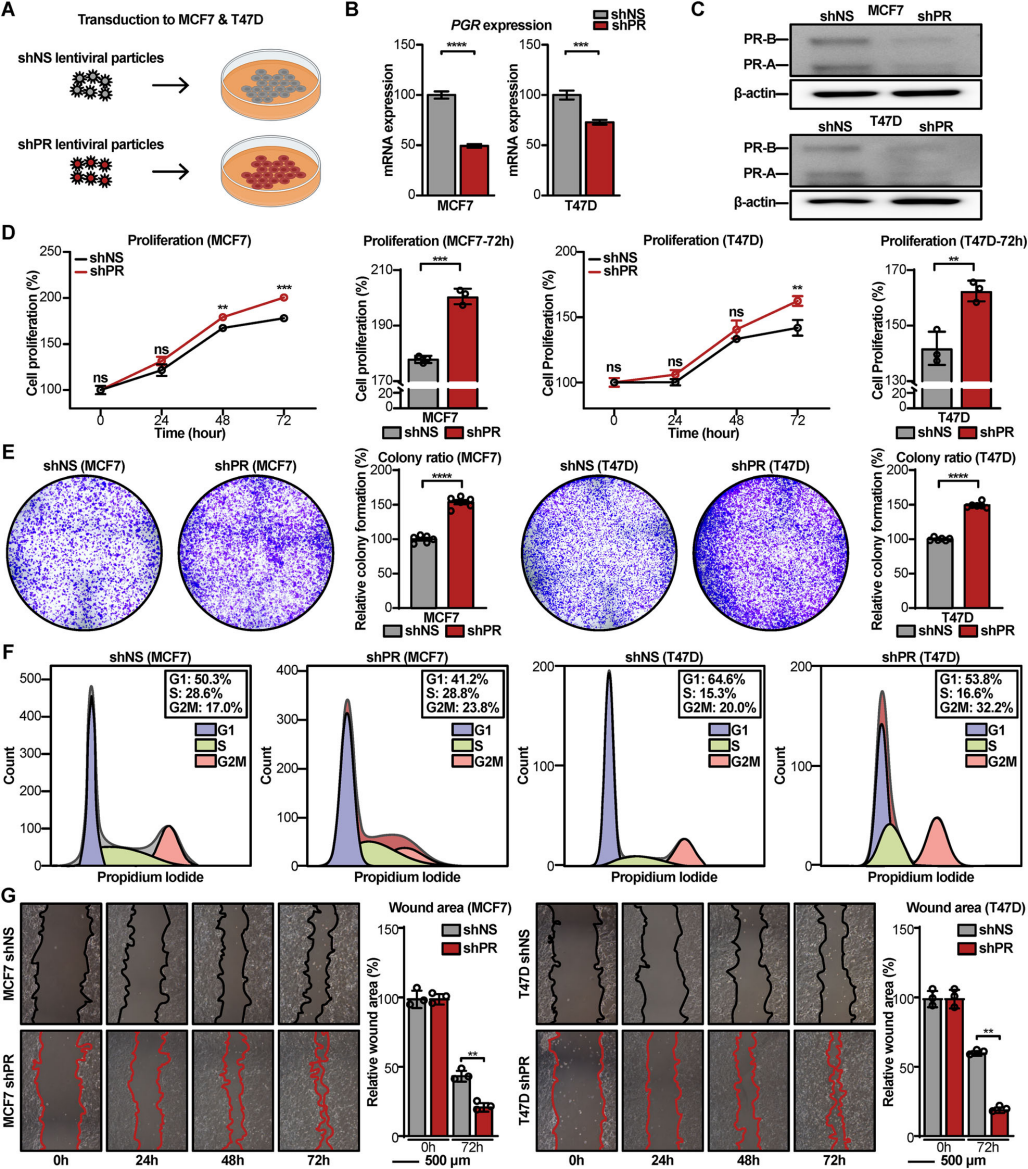
PR KD promotes aggressive phenotypes in luminal breast cancer cells. **A** Schematic model illustrating transduction of lentiviral particles to the luminal breast cancer cells, MCF7 and T47D. **B**, **C** PR KD was confirmed at the mRNA level by qPCR and the protein level by immunoblotting. **D** Cell proliferation assays showing that PR KD significantly increases the growth rate of both MCF7 and T47D cells. **E** Clonogenic assays demonstrating that PR KD cells form more and larger colonies compared to control cells, indicating enhanced long-term proliferative capacity. **F** Flow cytometry analysis of cell-cycle distribution showing that PR KD leads to a decreased proportion of cells in the G1 phase and an increased proportion in the S and G2/M phases. **G** Wound-healing assays demonstrating that PR KD promotes cell migration in MCF7 and T47D cells, as indicated by accelerated wound closure relative to control cells. For **B**, **D**, **E**, **G**, data are presented as mean ± SD. Statistical significance was assessed using an unpaired two-tailed t-test. * *p* < 0.05, ** *p* < 0.01, *** *p* < 0.001, **** *p* < 0.0001.

The relationship between PR and G6PD established by scRNA-seq analysis and clinical data analysis led to PR KD on the luminal breast cancer cell lines. Two luminal breast cancer cell lines, MCF7 and T47D [38, 39], were employed and lentiviral particles were used to silence *PGR* expression (Fig. 4A). PR reduction of PR KD cell lines (shPR) was confirmed by quantitative polymerase chain reaction (qPCR) and immunoblotting compared to control cell lines (shNS) (Fig. 4B and C).

Subsequently, to determine the functional consequences of PR KD, the proliferation rates of shNS and shPR were assessed. Notably, shPR exhibited a significantly higher proliferation rate compared to shNS (Fig. 4D). This increase was observed consistently in both MCF7 and T47D cell lines at 72 h, with statistical significance (MCF7: *p* = 0.0002; T47D: *p* = 0.0071). To further confirm the long-term proliferative capacity, a clonogenic formation assay was performed. The shPR formed a greater number of colonies compared to shNS, indicating enhanced tumorigenic potential (MCF7: *p* < 0.0001; T47D: *p* < 0.0001) (Fig. 4E, Fig. S7A).

To further evaluate the functional consequences of PR KD, a wound-healing assay was performed to assess the migratory capacity of the cells. The results demonstrated that PR KD cells exhibited significantly enhanced migratory motility compared to control cells (MCF7: FC = 1.92, *p* = 0.0014, T47D: FC = 3.44, *p* < 0.0001), as quantified by time-lapse images (Fig. 4G). These findings collectively indicate that PR loss promotes both proliferative and migratory behaviors, supporting an aggressive phenotype in luminal breast cancer cell lines.

To assess whether the proliferative phenotype induced by PR KD is reversible, PR KD cells were transfected with PR-A and PR-B expression plasmids (Fig. S8A and B). However, restoration of PR did not significantly alter proliferation compared with PR KD cells (Fig. S8C), indicating that this proliferative phenotype is not rescued by PR re-expression.

To understand the mechanism behind the increased proliferation, cell cycle analysis using propidium iodide (PI) was conducted. The shPR displayed a higher proportion of cells in the S and G2/M phases, with a concurrent reduction in the G1 phase, as determined by flow cytometry (Fig. 4F).

In breast cancer cells, factors that increase cancer proliferation imply a risk of high tumor grade, metastasis, and death induction in patients [40–43], suggesting that malignancy was induced in PR KD cells.

### Bulk RNA-seq analysis showed increased PPP activity in PR KD cells

**Fig. 5:**
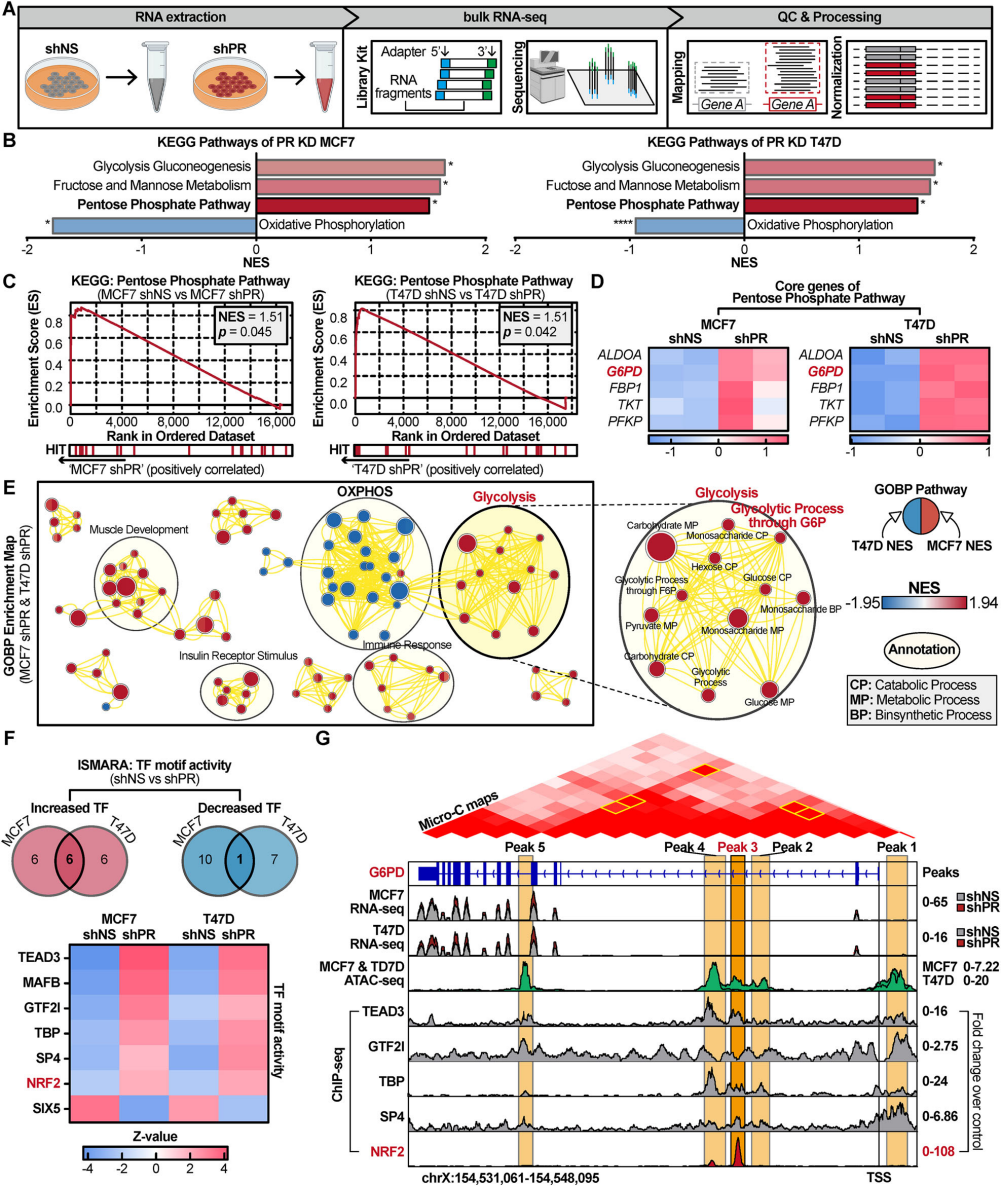
PR KD induces a metabolic shift toward glycolysis and the PPP, potentially involving NRF2-mediated G6PD upregulation. **A** Schematic diagram illustrating the experimental design for bulk RNA-seq of shNS and shPR. **B** GSEA summary plot showing significantly enriched KEGG pathways in PR KD cells. Pathways related to glucose metabolism (glycolysis, PPP) are upregulated, while OXPHOS is downregulated. **C** GSEA enrichment plots showing significant positive enrichment of the PPP in both MCF7 and T47D PR KD cells. **D** Heatmap displaying the expression levels of PPP-core genes from the GSEA analysis, with *G6PD* highlighted as a key gene consistently upregulated in both cell lines. **E** Enrichment map visualizing the landscape of altered biological processes. The map shows a clear separation between downregulated OXPHOS-related gene sets and upregulated glycolysis-related gene sets. **F** ISMARA analysis identifying key transcription factors (TF) regulated by PR KD in both cell lines. The heatmap displays the Z-scores of the most significantly activated and repressed TF, with NRF2 highlighted as a consistently activated factor. **G** Integrative analysis of the G6PD locus using publicly available ATAC-seq, Micro-C, and ChIP-seq data for key transcription factors. The tracks show specific binding of NRF2 at a putative enhancer element (Peak3), which physically interacts with the G6PD promoter, as confirmed by Micro-C data.

To research the transcriptomic changes of aggressiveness induced by PR KD in both cell lines, bulk RNA-seq was conducted. The sequencing was performed on Illumina NovaSeq 6000 platform system comparing shNS and shPR (Fig. 5A). The sequencing results confirmed no major differences in overall expression distribution between the two conditions (Fig. S9A). However, PCA revealed distinct transcriptomic profiles, indicating substantial transcriptional changes induced by PR KD (Fig. S9B).

As expected, *PGR* expression was significantly decreased in the shPR based on TPM values from bulk RNA-seq (Fig. S9C), verifying the knockdown efficiency. In addition, *ESR1* expression, a key target of tamoxifen therapy [44], was assessed (Fig. S9C). In MCF7 cells, there was no statistically significant difference between shNS and shPR. Similarly, in T47D cells, the fold change of –1.1 was not considered biologically or statistically meaningful, indicating that PR KD did not affect *ESR1* expression in either cell line (Fig. S9C).

Then, differentially expressed genes (DEG) analysis was performed to identify genes with significant expression differences between the two groups. Genes with a fold change ≥2.0 and *p* < 0.05 were defined as DEGs. In MCF7 cells, 227 genes were upregulated and 480 genes were downregulated, while T47D cells showed 536 upregulated and 168 downregulated genes (Fig. S9D). We then compared the DEG lists from both cell lines and found a limited overlap of only 53 genes (Fig. S9E–G). Of these shared genes, 39 genes were upregulated, and 14 genes were downregulated in both cell lines. Due to the limited overlap, conventional analysis was not feasible, leading to the application of GSEA using the full transcriptomic expression data.

GSEA based on KEGG pathways revealed a significant enrichment of metabolic pathways, including Glycolysis/Gluconeogenesis, Fructose and Mannose Metabolism, and the PPP, alongside a significant negative enrichment for Oxidative Phosphorylation (OXPHOS) in both shPR (Fig. 5B, Fig. S10A and B). This reduction in OXPHOS activity, potentially leading to impaired ATP production, may reflect a metabolic adaptation wherein PR KD breast cancer cells increasingly rely on glycolysis and the catabolism of glucose, fructose, and mannose to meet their energetic demands.

The most important aspect of GSEA results was the enrichment of the PPP in both MCF7 (NES = 1.51, *p* = 0.045) and T47D (NES = 1.51, *p* = 0.042) (Fig. 5C). Multiple core genes of PPP were identified in each cell line, and among these, *ALDOA*, *G6PD*, *FBP1*, *TKT*, and *PFKP* were common to both MCF7 and T47D (Fig. 5D). G6PD, the pivotal enzyme of the PPP, emerged as the sole shared core gene, highlighting its central role in PR KD-driven metabolic reprogramming.

To gain insight into the intracellular mechanisms underlying the PPP, we used the Enrichment Map application [45] based on gene ontology: biological process (GOBP) [46]. The enrichment map result was processed with *p*-value cutoff (*p* < 0.05), annotated using the AutoAnnote application [47], and visualized using Cytoscape [48]. The Enrichment map result showed that PR KD led to a decrease in OXPHOS and an increase in glycolysis consistent with GSEA-KEGG analysis (Fig. 5E). The map illustrated a clear metabolic shift, with a large cluster of downregulated OXPHOS-related processes and a distinct upregulated cluster related to glycolysis, which was connected to the G6PD-mediated PPP (Fig. 5E).

Taken together, these transcriptomic analyses unequivocally demonstrate that the PR KD induces profound metabolic reprogramming in luminal breast cancer cells. The coordinated upregulation of glycolysis, fructose/mannose metabolism, and the PPP, coupled with the concurrent downregulation of OXPHOS, signifies a decisive metabolic shift. This reprogramming moves the metabolic state of cells away from efficient ATP production via OXPHOS and towards a state optimized for rapid biosynthesis and redox defense, a hallmark of aggressive cancer phenotypes. Crucially, our analysis pinpoints G6PD as the central node in this PR-driven metabolic reprogramming, suggesting its pivotal role in sustaining this newly acquired metabolic state.

### NRF2 as a putative G6PD regulator in PR KD cells

The consistent upregulation of PPP, particularly G6PD, raised the question of which transcription factors mediate this metabolic reprogramming. To address this, we used the Integrated Motif Activity Response Analysis (ISMARA) [49] to predict transcription factor activities from our bulk RNA-seq data. This analysis identified 12 activated and 12 repressed transcription factors in PR KD MCF7, and 11 activated and 8 repressed factors in PR KD T47D (Fig. 5F). Six transcription factors, TEAD3, MAFB, GTF2I, TBP, SP4, and NFE2L2 (the gene encoding NRF2), were consistently activated in both cells, while only SIX5 was commonly repressed (Fig. 5F, Fig. S11A and B).

To investigate the chromatin landscape, we integrated our transcriptomic data with ATAC-seq datasets from MCF7 (GSE201262) [50] and T47D (GSE202511) [51] cells, along with Micro-C maps [52] to delineate 3D genome contacts (Fig. 5G). Due to the absence of chromatin immunoprecipitation sequencing (ChIP-seq) for MAFB in public data, subsequent binding analyses focused on the remaining five activated transcription factors. ChIP-seq fold-change-over-control signal tracks of ENCODE for TEAD3 (ENCFF464BRV; 0-16), GTF2I (ENCFF656XZC; 0-16), TBP (ENCFF576NZL; 0-24), SP4 (ENCFF630ZTF; 0–6.86), and NFE2L2/NRF2 (ENCFF690PPG; 0-108) were mapped onto the G6PD locus [53].

This integrated analysis revealed five putative regulatory elements (Peak1–Peak5) within the G6PD locus, defined by ATAC-seq peaks. Micro-C data confirmed strong chromatin interactions among all five elements, including long-range contacts between the most distal sites (Peak1 and Peak5), suggesting the formation of a regulatory hub. Peak1, located upstream of the G6PD transcription start site (TSS), likely functions as the promoter and showed enriched ChIP-seq signals for GTF2I and SP4. Peaks 2–5 represent potential distal enhancers, with TEAD3 and TBP binding predominantly at Peak4.

In striking contrast to the other factors, NRF2 displayed a highly specific and robust binding signal exclusively at Peak3. Given that NRF2 is a master regulator of the cellular response to oxidative and electrophilic stress, its specific recruitment to a G6PD regulatory element strongly suggests a stress-responsive mechanism driving G6PD upregulation. Consistent with this locus-specific recruitment, GSEA using the Transcription Factor Targets database revealed a significant upregulation of NRF2 target genes in PR KD cells (MCF7: NES = 1.4562, FDR = 0.040; T47D: NES = 1.5148, FDR = 0.021), supporting a broader activation of the NRF2 transcriptional program (Fig. S11C).

### PR KD induces a metabolic shift to glycolysis and enhances PPP-mediated antioxidant capacity

**Fig. 6:**
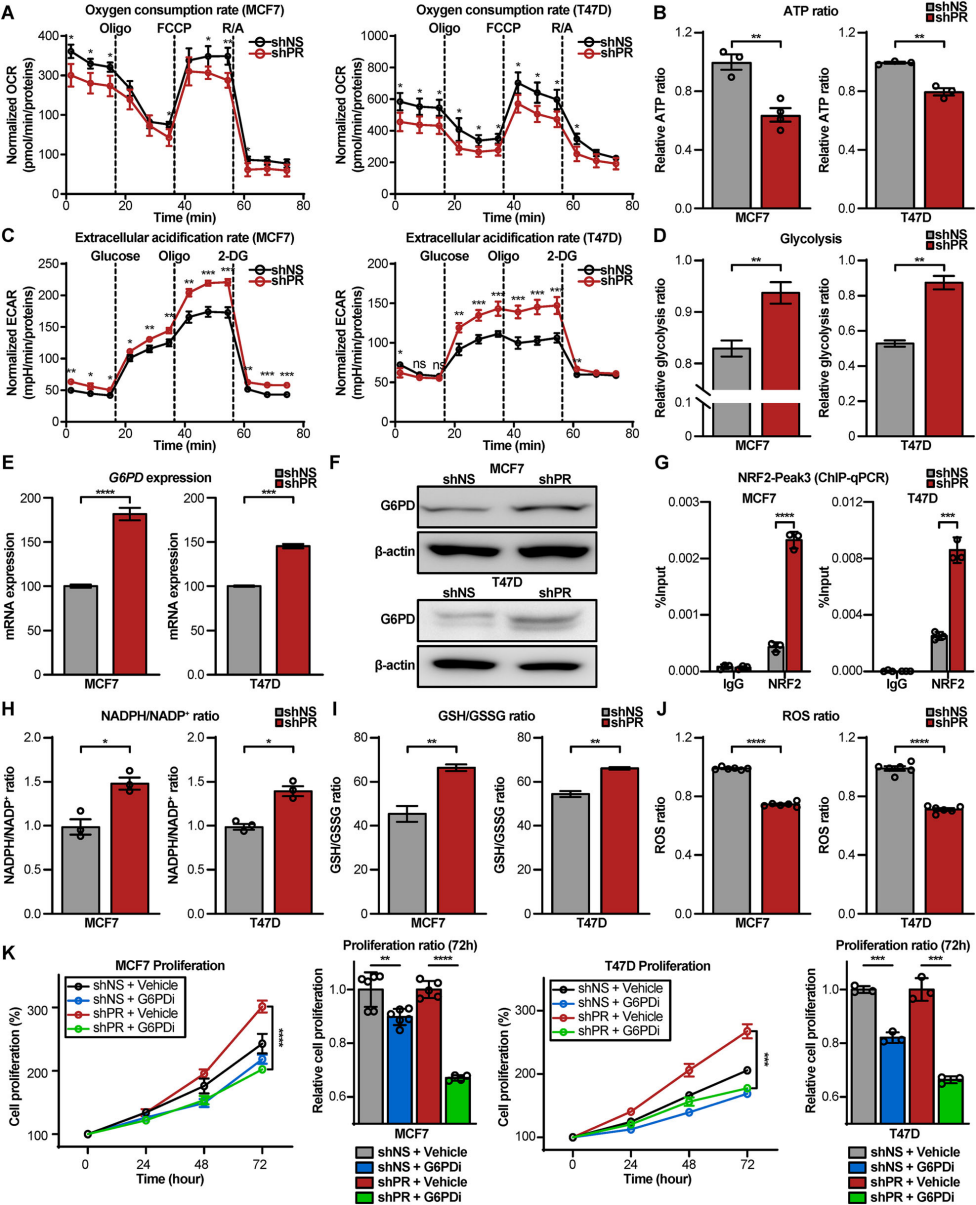
Functional validation of metabolic reprogramming and therapeutic targeting of the G6PD dependency in PR KD cells. **A**–**D** PR KD induces a metabolic shift from OXPHOS to glycolysis. **A**, **B** Mitochondrial respiration is decreased, shown by reduced oxygen consumption rate (OCR) and lower ATP production in PR KD cells. **C**, **D** Glycolysis is increased, shown by elevated extracellular acidification rate (ECAR) and higher glycolytic activity in PR KD cells. **E**, **F** Upregulation of G6PD confirmed at the mRNA level by qPCR and the protein level by immunoblotting. **G** ChIP-qPCR analysis showing significant enrichment of NRF2 binding at the G6PD enhancer element (Peak3) in PR KD cells. **H**–**J** Heightened PPP activity in PR KD cells enhances antioxidant capacity, as demonstrated by an increased NADPH/NADP⁺ ratio and GSH/GSSG ratio, and a corresponding decrease in intracellular ROS. **K** The hyperproliferative phenotype of PR KD cells is dependent on G6PD. Proliferation assay showing that PR KD cells are significantly more sensitive to inhibition by a G6PD inhibitor (G6PDi) compared to control cells. Data are presented as mean ± SD. Statistical significance was assessed using an unpaired two-tailed t-test. * *p* < 0.05, ** *p* < 0.01, *** *p* < 0.001, **** *p* < 0.0001.

To functionally validate the transcriptomic findings, key metabolic and molecular features were assessed in PR KD cells. Consistent with the GSEA results, PR KD cells displayed a significant reduction in mitochondrial respiration, as evidenced by decreased oxygen consumption rates (OCR) in a Seahorse XF analysis (Fig. 6A). This reduced OXPHOS led to a corresponding decrease in cellular ATP production (Fig. 6B). Concurrently, PR KD cells exhibited a metabolic shift towards glycolysis, showing significantly increased extracellular acidification rates (ECAR) and elevated glycolytic activity in a Seahorse XF analysis (Fig. 6C and D). These data confirm that PR loss drives a profound metabolic reprogramming from OXPHOS to glycolysis. Importantly, the glycolytic shift observed in PR KD cells is also consistent with the activation of NRF2, as several upregulated glycolytic enzymes (*ENO1*, *GAPDH*, and *ALDOA*) represent canonical NRF2 target genes (Fig. S11C). This suggests that NRF2 not only enhances antioxidant defense through PPP activation, but also contributes to glycolytic reprogramming, thereby coordinating a metabolic adaptation that supports both bioenergetic demands and redox homeostasis in PR-deficient cells.

Next, we examined whether the PPP is upregulated and directly regulated by NRF2. As predicted by the RNA-seq data, G6PD expression was significantly increased at both the mRNA and protein levels in shPR (Fig. 6E and F). Interestingly, despite the marked upregulation of G6PD, the total protein level of NRF2 remained unchanged upon PR KD (Fig. S12A), suggesting that NRF2 activation occurs independently of its transcriptional induction. In line with this, RNA-seq analysis revealed an increase in *KEAP1* expression (MCF7: FC = 1.25, FDR = 0.003; T47D: FC = 1.26, FDR = 2.2E-11) and cell line–specific variations in *CUL3* (MCF7: FC = 1.05, FDR = 0.532; T47D: FC = −1.09, FDR = 0.0029) (Fig. S12B). These transcriptional changes suggest possible modulation of the KEAP1–CUL3–NRF2 axis, possibly contributing to NRF2 stabilization. This prompted us to examine whether NRF2 binding to its target gene loci was enhanced as a functional consequence of its activation.

To experimentally confirm that NRF2 directly occupies the G6PD locus, chromatin immunoprecipitation followed by qPCR (ChIP-qPCR) was performed. Our multi-omics analysis previously identified Peak3 as the putative NRF2 binding site (Fig. 5F). Importantly, the presence of a canonical NRF2 binding motif within the Peak3 region is supported by a highly significant p-value (*p* = 1.26E-06), as predicted by the JASPAR 2024 database (Fig. S12C and D), underscoring the likelihood of direct NRF2 regulation. Therefore, primers for ChIP-qPCR were specifically designed to amplify the segment of Peak3 encompassing this NRF2 motif. Consistent with our integrative analysis, a significant enrichment of NRF2 binding at this precise regulatory element of the G6PD locus was observed specifically in PR KD cells (Fig. 6G). This finding provides direct experimental evidence that NRF2 occupies the G6PD locus via a binding motif, demonstrating direct NRF2 occupancy between PR silencing, NRF2 activation, and G6PD upregulation.

To further elucidate the temporal dynamics underlying NRF2 activation, we transiently silenced PR using siRNA and monitored time-dependent changes in PR and G6PD expression. qPCR analysis showed a gradual decline in PR expression following siRNA transfection, reaching approximately 0.60–0.68-fold in MCF7 and 0.69–0.85-fold in T47D cells at 8–10 h (Fig. S13A). Notably, G6PD expression exhibited an early transient increase, peaking at 10 h in MCF7 (1.90-fold) and 8 h in T47D (1.97-fold) relative to control cells (Fig. S13B), indicating a rapid and dynamic transcriptional response to transient PR KD. This early and transient upregulation of G6PD highlights its role as a rapid responder to PR depletion, reinforcing its importance in initiating antioxidant defense mechanisms during the initial phase of cellular adaptation.

To capture earlier redox alterations that might precede this transcriptional response, intracellular reactive oxygen species (ROS) levels were measured at shorter time intervals in transient PR KD. In MCF7 cells, ROS levels peaked at 2 h (1.31-fold), while T47D cells showed a similar pattern, with ROS peaking at 2 h (1.53-fold) (Fig. S13C). Interestingly, ROS levels subsequently declined to near- or even below-baseline levels by 8–48 h. Collectively, these findings delineate a temporal cascade in which acute PR depletion rapidly induces oxidative stress, which in turn acts as an upstream trigger for NRF2 activation and subsequent G6PD induction as a compensatory antioxidant mechanism.

To determine whether the NRF2–G6PD activation induced by PR loss is reversible, we performed PR rescue experiments by re-expression of PR in PR KD cells. Interestingly, re-expression of PR did not significantly restore G6PD levels, as assessed at both the mRNA and protein levels (Fig. S8A and B). This lack of reversal was mirrored at the regulatory level, where ChIP-qPCR showed no substantial reduction in NRF2 occupancy at the Peak3 region upon PR rescue (Fig. S14A). These results suggest that while PR loss initiates NRF2 activation and subsequent G6PD upregulation, this metabolic reprogramming may be sustained through irreversible or semi-permanent mechanisms, such as epigenetic modifications or persistent redox feedback loops, independent of PR restoration.

Functionally, the increased G6PD expression translated to heightened PPP activity. The intracellular NADPH/NADP⁺ ratio, a direct readout of PPP flux, was significantly elevated in PR KD cells (Fig. 6H). This enhanced NADPH production contributed to a greater antioxidant capacity, as demonstrated by an increased GSH/GSSG ratio (Fig. 6I) and a corresponding reduction in intracellular ROS levels (Fig. 6J). Taken together with the temporal and mechanistic analyses described above, these findings show that PR KD cells undergo NRF2-mediated metabolic rewiring that enhances PPP-driven NADPH production and strengthens their antioxidant defense capacity.

### The aggressive phenotype of PR KD cells is dependent on G6PD and can be targeted by G6PDi

Given that PR KD cells exhibit heightened PPP activity, we next sought to determine if this metabolic rewiring creates a targetable dependency. To this end, the effect of pharmacologically inhibiting PPP was assessed using G6PDi, a selective small-molecule inhibitor of G6PD [19]. As the rate-limiting enzyme, G6PD inhibition effectively blocks PPP flux and depletes the intracellular NADPH pools that are critical for proliferation and antioxidant defense [19].

The anti-proliferative effect of G6PDi was compared between shNS and shPR. While G6PDi treatment only modestly affected the growth of shNS, it caused a profound reduction in the proliferation of shPR in both MCF7 (*p* < 0.0001) and T47D (*p* = 0.0002) (Fig. 6K). This result demonstrates that the hyperproliferative state of PR-low cells is critically dependent on G6PD activity, highlighting a key therapeutic vulnerability.

Furthermore, the potential for a combination therapy was explored with tamoxifen, a standard-of-care endocrine therapy for luminal breast cancer. A greater reduction in proliferation was observed when G6PDi was combined with tamoxifen compared to the effects of each drug administered individually (Fig. S15A). These results suggest a potential synergistic interaction between G6PDi and tamoxifen, presenting a promising therapeutic strategy to overcome resistance and improve outcomes for patients with PR-low luminal breast cancer.

In summary, PR loss in luminal breast cancer cells drives a stable metabolic reprogramming toward heightened PPP flux via NRF2‑mediated G6PD transcription, enhancing NADPH and glutathione redox capacity to support an aggressive phenotype. This state creates a specific vulnerability to G6PD inhibition, as evidenced by the marked sensitivity of PR‑low cells to G6PDi, particularly when combined with tamoxifen.

**Fig. 7:**
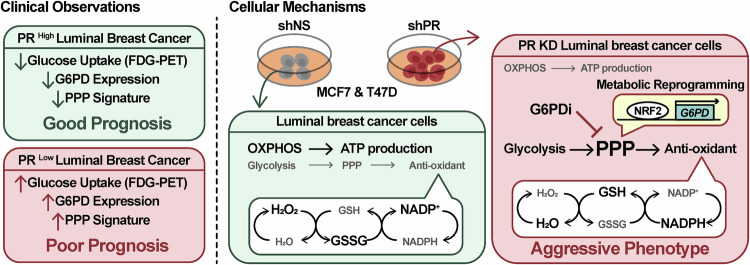
A proposed model for PR-loss-driven metabolic reprogramming and therapeutic targeting in luminal breast cancer. This model contrasts the clinical observations in patients with the underlying cellular mechanisms. Left (Clinical Observations): PR expression status defines distinct clinical phenotypes. Low PR expression is associated with an aggressive profile characterized by a cascade of observable features: increased Glucose Uptake (FDG-PET), elevated G6PD Expression, and an activated PPP Signature. This combination of metabolic and molecular markers is ultimately linked to a Poor Prognosis. Conversely, high PR expression is characteristic of a less aggressive phenotype with reduced levels of these markers, leading to a Good Prognosis. Right (Cellular Mechanisms): Mechanistically, loss of PR leads to NRF2 activation, which directly binds to the G6PD locus to induce its transcription. The resulting G6PD upregulation enhances PPP flux, increasing the NADPH/NADP⁺ and GSH/GSSG ratios to strengthen antioxidant capacity. This metabolic rewiring supports tumor aggressiveness and establishes a critical dependence on G6PD, which can be therapeutically exploited using G6PD inhibitors to suppress tumor growth.

Together with clinical correlations linking low PR expression to elevated glucose uptake, high G6PD expression, and poor prognosis, these findings converge into a unifying model (Fig. 7) that illustrates both the mechanistic basis and therapeutic potential of targeting the PR‑loss-G6PD axis in luminal breast cancers.

## Discussion

In this study, we elucidate a key mechanism underlying the aggressive phenotype of luminal breast cancers with low PR expression. We demonstrate that PR loss drives a metabolic shift towards the PPP, which in turn supports heightened cancer cell proliferation. Critically, this aggressive phenotype depends on G6PD, the pivotal enzyme of PPP, and it can be attenuated by pharmacological inhibition. These findings identify a targetable metabolic vulnerability in PR-low tumors and propose a therapeutic strategy of using G6PD inhibitors for patients selected by PR status as a biomarker.

PR is essential for regulating the activity of estrogen receptors α (ERα) in breast cancer [54]. As an upregulated target gene of ER, PR expression largely depends on estrogen levels. The mechanism regarding the prognostic effect of PR in breast cancer is subject to various opinions. Previous study has suggested that PR directly inhibit estrogen-induced tumor growth by translocating ER from mitotic sites to apoptosis and cell death genes [55]. Additionally, another study claimed that PR regulated overall tumor growth by modulating RNA polymerase III [56]. It was also reported that ER-positive/PR-negative breast cancer exhibits upregulation of PI3K/Akt/mTOR pathway activity compared to PR-positive luminal breast cancer [57]. On the other hand, based on our previous clinical studies [11, 12], we focused on the association between PR and glucose metabolism, and we were able to obtain largely positive results.

To study the significance of PR expression in luminal breast cancer, we performed scRNA-seq analysis on a published patient dataset. We found that proliferative luminal epithelial cells with low PR expression were associated with increased PPP activity and G6PD expression. These findings indicated that proliferative cells with lower PR expression might be associated with increased PPP activity, driven by a heightened demand for NADPH, suggesting metabolic shift based on PR expression. G6PD, the rate-limiting enzyme of the PPP, is crucial for generating NADPH, a key cofactor required for maintaining redox homeostasis and supporting cellular metabolic processes. The correlation between low PR expression and increased G6PD activity suggests that metabolic reprogramming may drive the aggressiveness of these luminal epithelial cells. These results highlight the aggressiveness of PR-low proliferative cells and point to G6PD as a potential therapeutic target.

To verify the clinical implications of our scRNA-seq analysis results, we devised and conducted analyses using various methods, including TCGA dataset [36], tumor tissue from luminal breast cancer patients in the Yonsei cohort, and the Kaplan–Meier plotter web tool [37]. As a result, through multiple analyses, we confirmed that PPP and G6PD expression were activated in PR-low tumors and that this activation was associated with poorer survival outcomes.

An important observation from our analysis of clinical cohorts is the notable heterogeneity in G6PD expression among patients with PR-low tumors. While our data establishes a strong association between low PR status and elevated G6PD/PPP activity, the existence of a PR ^Low^/G6PD ^Low^ subgroup suggests that PR loss alone is not sufficient to induce G6PD expression. Instead, we propose a more nuanced model wherein the loss of PR acts as a key sensitizing event, creating a permissive state for G6PD upregulation.

In this model, the ultimate level of G6PD expression is likely determined by the activity of additional regulatory factors that converge on the G6PD gene. Our mechanistic data strongly points to NRF2, the master transcriptional regulator orchestrating cellular antioxidant and metabolic responses [18], as the central mediator in this process. Therefore, we speculate that the variability in G6PD levels within the PR-low population could be attributed to patient-specific differences in the magnitude of NRF2 activation.

This, in turn, may be influenced by factors such as the degree of oxidative stress in the tumor microenvironment or the presence of other co-existing genetic and epigenetic alterations. This refined model, involving both PR loss and subsequent NRF2-activating signals, better reconciles our clinical and mechanistic findings and underscores the complex, multi-faceted nature of metabolic reprogramming in luminal breast cancer.

Given the reports that higher G6PD expression levels are associated with a higher hazard ratio in breast cancer patients [17], we hypothesized that PR expression might influence luminal breast cancer in association with G6PD. MCF7 and T47D cells were transduced with lentiviral particles encoding PR-targeting shRNA, and silencing efficiency was confirmed by Western blot analysis. These PR KD cells exhibited faster proliferation rates, increased PPP activity, and elevated G6PD expression compared to control cells. In addition, wound-healing assays revealed that PR KD cells displayed significantly enhanced migratory capacity relative to control cells, further indicating that PR loss not only promotes metabolic and proliferative advantages but also enhances migratory capacity, thereby conferring a more aggressive phenotype.

A key finding of our work is the elucidation of the specific mechanistic cascade linking PR loss to aggressive phenotype. We demonstrate that the upregulation of G6PD and the PPP in PR-low cells is not a direct effect but is instead mediated by the transcription factor NRF2. Our multi-faceted approach, combining unbiased bioinformatic analysis (ISMARA) with integrative multi-omics and experimental validation, pinpointed NRF2 as the central mediator. We showed that PR KD leads to a significant increase in NRF2’s binding to a specific enhancer element within the G6PD locus, thereby driving its expression. Intriguingly, this enhanced activity occurred without a corresponding increase in total NRF2 protein levels, suggesting that PR loss triggers a post-translational activation of NRF2, likely through mechanisms such as protein stabilization.

Importantly, our temporal analysis using transient siRNA-mediated PR KD revealed that acute PR depletion rapidly triggered a short-lived oxidative burst. This early ROS surge might precede the induction of G6PD expression, supporting the notion that oxidative stress serves as an upstream signal for NRF2 activation. Indeed, such transient oxidative events have been well established to promote NRF2 stabilization and nuclear translocation through oxidation of KEAP1 cysteine residues, leading to transcriptional activation of canonical antioxidant targets [18]. Thus, the observed sequence supports a model in which PR loss initiates a redox-mediated NRF2 activation cascade that reprograms cellular metabolism toward the PPP to restore redox homeostasis. However, transient PR KD using siRNA did not sustain G6PD expression, suggesting that additional regulatory components may be required to maintain prolonged NRF2–G6PD activation.

Next, our findings highlight important translational implications. GSEA analysis further indicated that some glycolysis-related genes are NRF2 targets. This finding is consistent with the observed increase in ECAR and the metabolic shift toward glycolysis in PR-low cells. Notably, clinical studies have also reported that PR-low breast cancers exhibit significantly higher FDG-PET SUV values [11, 12], further supporting the link between PR loss, glycolytic reprogramming, and aggressive tumor metabolism. This discovery of the PR-NRF2-G6PD axis establishes a mechanistic framework for understanding how hormone receptor status can dictate the metabolic phenotype of cancer cells.

Having established the mechanistic link, we next sought to determine whether this reprogramming is reversible upon PR restoration. Re-expression of PR in PR KD cells did not reverse the proliferative phenotype, as indicated by sustained growth rates comparable to those observed in PR KD cells. At the molecular level, PR restoration also did not attenuate NRF2-driven G6PD upregulation, with both mRNA and protein levels remaining elevated. Together, these findings indicate that PR loss acts primarily as an initiator of NRF2 activation and subsequent metabolic reprogramming [58], triggering a cascade that becomes self-sustaining and independent of continued PR suppression. This persistent state may be maintained through epigenetic mechanisms [59], including histone modifications and DNA methylation at key regulatory loci, which stably maintain NRF2 target genes in an active state even after the initial stimulus has been withdrawn.

The therapeutic implication of this newly identified axis is significant. Treatment with the G6PD inhibitor, G6PDi, markedly suppressed the proliferation of PR KD cells compared to control cells. This supports a functional link between PR expression and G6PD-driven metabolic reprogramming. The observed reduction in proliferation highlights the potential of G6PD as a therapeutic target, especially in aggressive PR-low luminal breast cancer cells. These results provide important information for understanding the interaction between PR and G6PD and suggest a new approach for the treatment of aggressive luminal breast cancer.

To our knowledge, this is the first study to experimentally demonstrate the functional correlation between PR and G6PD. Increased expression of G6PD has been consistently reported in various cancers, including renal cell carcinoma, gastric cancer, and bladder cancer [60–62]. It has been also reported that G6PD expression is associated with poor prognosis in breast cancer [17]. However, previous studies did not establish a connection between PPP activity and PR expression, whereas our work identifies and validates a direct association between PR loss and G6PD upregulation.

G6PD inhibitors have not yet been used in the treatment of malignant tumors in clinical practice, but research on the use of G6PD inhibitors is currently under investigation for various cancers such as ovarian cancer and tongue cancer [63, 64]. In our study, we also validated the therapeutic potential of G6PD inhibition in luminal breast cancer and identified PR expression as a potential biomarker to guide the clinical application of G6PD inhibitors.

Several limitations should be noted. First, our mechanistic and functional analyses primarily relied on two luminal breast cancer cell lines (MCF7 and T47D). While these are well-established models for ER + /PR+ breast cancer, they do not fully recapitulate the molecular and phenotypic heterogeneity of cancer, including resistant or metastatic variants. Second, our findings are based on in vitro assays, and in vivo validation using xenograft or genetically engineered mouse models will be essential to confirm the efficacy and safety of targeting the PR-NRF2-G6PD axis in a more physiologic context. Third, although our results showed strong concordance with large-scale patient datasets, these correlations are retrospective, and prospective validation in independent cohorts will be required. Fourth, while our temporal analyses suggest that ROS may act upstream of NRF2 to influence G6PD induction, the precise causal relationship remains to be fully elucidated. Finally, our study focused primarily on glycolysis and the PPP, but other metabolic pathways such as glutamine or lipid metabolism may also be rewired in PR-low states, and their contribution remains to be investigated. Taken together, further work across diverse models and clinical specimens will be necessary to fully establish the generalizability and translational potential of our findings.

In conclusion, we propose a therapeutic strategy for luminal breast cancer based on PR expression as a biomarker. Our findings indicate that the aggressiveness of PR-low tumors is closely linked to metabolic reprogramming through the PPP and its key enzyme, G6PD, highlighting this axis as a compelling therapeutic vulnerability in luminal breast cancer.

## Materials and methods

### scRNA-seq analysis

Single-cell RNA-sequencing data from luminal breast cancer patients were obtained from GEO datasets GSE161529 [27] and GSE176078 [28]. The raw count matrices were processed using the Seurat package (v4.3.0) in R (v4.1.3). Initial quality control was performed by filtering out cells with fewer than 1000 detected genes. The data was then normalized using the ‘LogNormalize’ method. To combine datasets, we used the ‘IntegrateData’ function, resulting in an integrated Seurat object comprising approximately 100,000 cells. PCA was performed using the ‘RunPCA’ function, and the top principal components were used for UMAP-based dimensionality reduction (‘RunUMAP’). Cell clustering was performed using the ‘FindNeighbors’ and ‘FindClusters’ functions, which identified eight distinct clusters. Visualization of the integrated data was carried out using ‘DimPlot’, ‘DotPlot’, ‘VlnPlot’, and ‘FeaturePlot’. The epithelial cell cluster was isolated for downstream analysis using the ‘subset’ function. Cell cycle scores were calculated using the ‘CellCycleScoring’ function. Gene set enrichment scores for the PPP were estimated using the escape package (v1.4.0) [65], providing normalized enrichment scores (NES) and false discovery rates (FDR). Finally, copy number variation (CNV) analysis was performed on the subsetted epithelial cells using the CopyKAT package (v1.1.0) [35].

### TCGA analysis

The clinical and mRNA sequencing data of TCGA: Breast Invasive Carcinoma Patients [36] was collected from cBioportal (https://www.cbioportal.org). Among the patients (*n* = 1084), luminal breast cancer patients (*n* = 499) were distinguished by subtype, which is clinical data. Then, using transcriptomic data from sequencing data, patients were stratified into PR ^Low^ and PR ^High^ groups based on the median expression of PGR. To confirm the transcriptomic phenotype differences, PCA was conducted based on transcriptomics data using FactoMineR package (v.2.8) and factoextra package (v.1.0.7) in R. The enrichment plot was visualized to present the NES and nominal *p*-value of PPP based on KEGG using GSEA (v.4.3.2).

### Kaplan–Meier analysis

Kaplan–Meier plotter web tool was employed to visualize the survival probability of luminal breast cancer patients based on expression levels of PR and G6PD [37]. The survival probability of Q1 and Q4 was compared between patients with PR and G6PD expression, as divided into quartiles. Furthermore, we compared the survival probability based on G6PD expression within each group: the low PR expression group (Q1) and the high PR expression group (Q4). To this end, the log-rank p-value and hazard ratio were calculated and presented.

### IHC

For breast cancer patients diagnosing and subtyping, ER, PR, and HER2 staining were evaluated by IHC using ER (1:100; Novocastra, clone 6F11), PR (1:100; Novocastra, clone 16) and HER2 (1:100; Ventana Medical Systems, clone 4B5) antibodies as previously described [66]. ER and PR status were determined by the modified Allred score and HER2 status was determined following to the American Society of Clinical Oncology (ASCO)/College of American Pathologists (CAP) guideline [67]. In accordance with the clinical data obtained, patients with hormone receptor-positive and HER2-negative were diagnosed with luminal breast cancer.

For G6PD staining, tissue microarray (TMA) paraffin blocks were prepared [66], using an Accu Max Array tissue-arraying instrument (Petagen Inc.). Then, each tissue microarray slide was stained with a G6PD (1:100; Novus Biologicals, NB100-236) antibody and counterstained with hematoxylin. After staining, the cytoplasmic-G6PD expression on each slide was scored by a pathologist (Yoon Jin Cha) using a light microscope (400x magnification). The results of IHC staining were scored as 0 (negative), 1+ (weak), 2+ (moderate), and 3+ (strong).

### Cell lines and silencing PR

The MCF7 and T47D cell lines were generously provided by the laboratory of Professor Sung Gwe Ahn. All cell lines tested negative for mycoplasma contamination. Both cell lines were cultured in RPMI 1640 (Corning, 10-041-CV) supplemented with 10% fetal bovine serum (FBS; Corning, 35-015-CV) and 1% penicillin-streptomycin (PS; Gibco, 15140-122) in a humidified 5% CO_2_ incubator at 37 °C. Cells were subcultured at approximately 75% confluence using 0.25% trypsin-EDTA (Gibco, 25200-072). For stable silencing of PR, cells were transduced with TRC-pLKO-U6 lentiviral particles encoding PR-targeting shRNA and selected with 1 µg/mL puromycin for 5 days to establish stable PR KD cell lines. For transient silencing, cells were transfected with 20 nM siRNA specific to PR (Horizon Discovery, L-003433-00-0005) or a non-targeting control (Horizon Discovery, D-001810-10-05) using Lipofectamine RNAiMAX (Thermo Fisher Scientific, 13778150) according to the manufacturer’s instructions. Cells were harvested at the indicated time points for subsequent assays.

### Real-time qPCR

Cellular total RNA was extracted using TRIzol reagent (Invitrogen, 15596018) according to the manufacturer’s protocol. Complementary DNA (cDNA) was established using ImProm-II Reverse Transcriptase (Promega, A3803). Real-time qPCR was performed using TOP Real qPCR 2X Pre-MIX (Enzynomics, RT501S) with specific primers on a CFX Connect Real-Time PCR System (Bio-Rad, 1855201). Gene expression levels were normalized to 36B4 as housekeeping gene using ΔΔCt method. The sequences of the primers used in the qPCR are shown below.

RPLP0 (36B4) (F: CGTCCTCGTTGGAGTGACA, R: CGGTGCGTCAGGGATTG)

PGR (F: ACCCGCCCTATCTCAACTACC, R: AGGACACCATAATGACAGCCT)

G6PD (F: CGAGGCCGTCACCAAGAAC, R: GTAGTGGTCGATGCGGTAGA)

NRF2-ChIP (F: GGGGAGTGCCAACATCATCA, R: GCCTCCCCTTTCCTCAGAAC)

### Immunoblot assay

Cellular proteins were extracted using lysis buffer (50 mM Tris-HCl, pH 8.0, 200 mM NaCl, 0.5% NP-40) with Xpert Protease Inhibitor Cocktail Solution (GenDEPOT, P3100-001). Concentration of protein was estimated using the BCA Protein Assay kit (Thermo Fisher Scientific, 23227). Equal amounts of protein were separated by sodium dodecyl sulfate polyacrylamide gel electrophoresis (SDS-PAGE) and transfer to polyvinylidene difluoride (PVDF; BIO-RAD, 162-0177). The PVDF membranes were blocked with 5% skim milk (Difco, 232100) and incubated with diluted antibodies at 4 °C for overnight. Then, it was probed with horseradish peroxidase conjugated secondary antibodies. The immunoblots were visualized using enhanced chemiluminescence solution (BIO-RAD, 1705061).

The primary antibodies against PR (1:2000; Santa Cruz Biotechnology, sc-166169), G6PD (1:2000; Cell Signaling Technology, 8866S), NRF2 (1:2000; Abcam, ab137550), and β-actin (1:5000; Santa Cruz Biotechnology, sc-47778) were used. The secondary antibodies against mouse IgG (1:10000; Cell Signaling Technology, 7076S) and rabbit IgG (1:10000; Abcam, ab6721) were used.

### Proliferation assay

Cell proliferation was measured using Cell Counting Kit-8 (CCK8; Dojindo, CK04-13) according to manufacturer’s instructions. Briefly, the CCK8 was treated with a volume of 1/10th the scale of the media and incubated for one hour at 37 °C. The absorbance was then measured at 450 nm using a spectrophotometer.

### Clonogenic assay

Cells were seeded into plates and cultured for approximately two weeks to allow colony formation. Colonies were fixed with 4% paraformaldehyde (Biosesang, PC2031-100-00) and washed with PBS. Subsequently, cells were stained with 0.5% crystal violet solution (Sigma-Aldrich, V5265-250ML). After staining, plates were rinsed twice with distilled water. Finally, colonies were solubilized using 10% acetic acid (Sigma-Aldrich, A6283-100ML) and absorbance was measured at 590 nm using a spectrophotometer.

### Wound-healing assay

Cells were seeded into 6-well plates and cultured for approximately one week until they reached near-confluency. A sterile 200 µL pipette tip was then used to create a uniform scratch across the monolayer, and detached cells were removed by gently washing with culture medium. Images of the scratched areas were captured at 24-h intervals to monitor cell migration. The wound area was quantified using ImageJ software (v.1.54p), and migration rates were calculated as the percentage of wound closure relative to the initial scratch area.

### Cell cycle assay

MCF7 and T47D cells were cultured in 100 mm dishes until they reached approximately 70% confluency. Cells were harvested, washed with PBS, and fixed with ice-cold 70% ethanol at −20 °C overnight. After fixation, the cells were washed again with PBS and resuspended in a PI staining solution containing 50 µg/mL PI, 50 µg/mL RNase A, and 0.1% Triton X-100 in PBS. Following a 30-min incubation in the dark at room temperature, the DNA content of the cells was analyzed by flow cytometry. The distribution of cells in G1, S, and G2/M phases was quantified using a flow cytometer, with PI fluorescence detected in the PI channel. The resulting data were analyzed using FlowJo software (BD Biosciences, v.10.7.1) to determine the percentage of cells in the G1, S, and G2/M phases of the cell cycle.

### Overexpression assay

MCF7 and T47D cells were seeded at a density of 2 × 10^6^ cells per 100 mm dish and cultured overnight. On the day of transfection, the culture medium was replaced with PS-free RPMI 1640 supplemented with 10% FBS.

For co-transfection, cells were transfected with 75 ng of a PR-A plasmid (Addgene, #89119) and 75 ng of a PR-B plasmid (Addgene, #89130). The transfection was performed using Lipofectamine^TM^ 3000 Transfection Reagent (Invitrogen, L3000015) according to the manufacturer’s protocol. After 6 h of incubation, the medium containing the transfection complexes was removed and replaced with complete growth medium. Cells were cultured for an additional 48 h before being harvested for subsequent analysis.

### Bulk RNA-seq analysis

Total RNA samples were extracted and transported to Macrogen Inc. (https://www.macrogen.com). Briefly, a library was established using TruSeq Stranded mRNA Library Prep Kit (Illumina) according to manufacturer’s instructions. Then, sequencing was performed by NovaSeq6000 (Illumina) platform using a NovaSeq 6000 S4 Reagent Kit (Illumina). Raw sequencing data were qualified using FastQC (v.0.11.7), trimmed using Trimmomatic (v.0.38), mapped using HISAT2 (v.2.1.0) and Bowtie2 (v2.3.4.1), and assembled using StringTie (v.2.1.3.b). Trimmed mean of M-value (TMM) normalization was conducted to normalize the read count value using edgeR package. And the DEGs were estimated using edgeR package.

### Gene set enrichment analysis (GSEA)

GSEA was performed to identify significantly enriched biological pathways using GSEA software (v4.3.3). For our in-house bulk RNA-sequencing data, normalized expression values were used as input. The analysis was conducted using the ‘Ratio_of_Classes’ metric for pairwise comparisons between experimental groups. For publicly available TCGA datasets, the ‘Signal2Noise’ metric was employed, which is suitable for large sample sizes and continuous data. In both analyses, the enrichment statistic was set to ‘weighted’, and all other parameters were maintained at their default settings. Gene sets were obtained from the Molecular Signatures Database. Given the exploratory nature of this analysis and the limited number of gene sets meeting the stringent FDR cutoff, we used a nominal *p* < 0.05 to identify potentially relevant pathways for further investigation.

### OXPHOS and glycolysis assay

The metabolic activity of the cells was assessed by measuring the OCR for mitochondrial respiration and the ECAR for glycolysis, using a Seahorse XFp Analyzer (Agilent Technologies). Cells were seeded in Seahorse XFp cell culture miniplates (XFp Fluxpak, Agilent, 103022-100) at a density of 1 × 10^4^ cells per well and cultured overnight.

Before the assay, the growth medium was replaced with the appropriate Seahorse XF RPMI assay medium (pH 7.4; Agilent, 103576-100) and cells were incubated in a non-CO2 incubator at 37 °C for 1 h. For the Mito Stress Test, the assay medium was supplemented with 10 mM glucose (Agilent, 103577-100), 2 mM L-glutamine (Agilent, 103579-100). For the Glycolysis Stress Test, the assay medium was supplemented only with 2 mM L-glutamine.

The assays were performed using the XFp Cell Mito Stress Test Kit (Cat. No. 103010-100; Agilent) and the Seahorse XFp Glycolysis Stress Test Kit (Cat. No. 103017-100; Agilent). For the Mito Stress Test, OCR was measured following sequential injections of Oligomycin (1.0 µM), FCCP (0.75 µM), and Rotenone/Antimycin A (1.0 µM). For the Glycolysis Stress Test, ECAR was measured following sequential injections of glucose (7.5 mM), oligomycin (1.0 µM), and 2-deoxyglucose (2-DG; 25 mM). After the assays, OCR and ECAR data were normalized to the total protein content per well, which was quantified using a BCA protein assay kit.

### ChIP assay

Cells were cross-linked with 1% methanol-free formaldehyde (Thermo Fisher Scientific, 28908) for 10 min at room temperature, and the reaction was quenched with 0.125 M glycine. Cell pellets were lysed in Nuclear Lysis Buffer (50 mM Tris-HCl pH 8.0, 5 mM EDTA, 150 mM NaCl, 0.5% NP-40), and the isolated nuclei were resuspended in Sonication Buffer (50 mM Tris-HCl pH 8.0, 10 mM EDTA, 1% SDS). Chromatin was sheared to an average fragment size of 200–500 bp using a Covaris M220 focused-ultrasonicator (Settings: Peak Power 75 W, Duty Factor 15%, 300 Cycles/Burst, 30 minutes).

The sheared chromatin was diluted 1:10 in Dilution Buffer (1% Triton X-100, 150 mM NaCl, 20 mM Tris-HCl pH 8.0, 2 mM EDTA), with 10% reserved as input. The remaining chromatin was pre-cleared with Protein A/G Sepharose beads (Cytiva, 17-0618-01; 17-5280-01) and then incubated overnight at 4 °C with an anti-NRF2 antibody or normal IgG (Cell Signaling Technology, 2729S).

Immune complexes were captured with Protein A/G beads and washed sequentially with Low Salt Wash Buffer (0.1% SDS, 1% Triton X-100, 2 mM EDTA, 150 mM NaCl, 20 mM Tris-HCl pH 8.0), High Salt Wash Buffer (same as Low Salt but with 500 mM NaCl), LiCl Wash Buffer (0.25 M LiCl, 1% NP-40, 1% deoxycholate, 1 mM EDTA, 10 mM Tris-HCl pH 8.0), and finally with TE buffer. Chromatin was eluted, treated with Proteinase K (QIAGEN,175042929), and reverse-cross-linked at 65 °C overnight. DNA was purified using the PCR Purification Kit (QIAGEN, 28106). Target DNA enrichment was quantified by qPCR, and sonication efficiency was confirmed by agarose gel electrophoresis.

### NADPH/NADP^+^ assay

The ratio of NADPH/NADP^+^ was measured using NADP/NADPH Quantitation Kit (Sigma-Aldrich, MAK038). Briefly, NADP/NADPH were extracted using extraction buffer and quantified levels of NADPH and total NADP following manufacturer’s instructions. The NADPH/NADP+ ratio was calculated as follows: NADPH / (total NADP − NADPH).

### GSH/GSSG assay

The ratio of GSH/GSSG was measured using GSH/GSSG-Glo^TM^ Assay Kit (Promega, V6611). Briefly, the levels of total glutathione (GSH + GSSG) and oxidized glutathione (GSSG) were quantified in separate luminescence-based reactions according to the manufacturer’s instructions. The final ratio was calculated as follows: (Total Glutathione - GSSG) / (GSSG/2).

### ROS assay

The production of intracellular ROS was detected using the fluorescent probe 2’,7’-Dichlorodihydrofluorescein diacetate (H2DCFDA; Thermo Fisher Scientific, D399). Briefly, cells were washed with PBS and then incubated with 10 µM H2DCFDA in serum-free medium for 30 min at 37 °C in the dark. After incubation, the fluorescence of the oxidized product, DCF, was measured using a fluorescence microplate reader at an excitation wavelength of 495 nm and an emission wavelength of 520 nm.

### Statistical analysis

All statistical analyses were performed using GraphPad Prism (v9.3) and R (v4.1.3). For comparisons between two groups, either the unpaired two-tailed Student’s *t* test or the Wilcoxon rank-sum test was used, depending on data distribution. Variance homogeneity was evaluated before applying parametric tests. Categorical variables were analyzed using the Chi-square test. Data are presented as mean ± SD or median, as appropriate based on distribution. All experiments were performed with at least three independent biological replicates unless otherwise specified. *p* < 0.05 were considered statistically significant.

## Supplementary information

Supplementary Figure

Uncropped Western Blots

## Data Availability

The RNA-seq data generated in this study have been deposited in the NCBI BioProject database under accession number PRJNA1308170.

## Code availability

Custom R scripts used for scRNA-seq analysis are available from the corresponding author upon reasonable request.
