# Supplementary material for: PR inhibition stimulates G6PD expression to enhance malignancy in luminal breast cancer
Source: Cell Death Dis. 2025 Dec 21;17(1):104. doi: 10.1038/s41419-025-08365-7 (PMC12847734; doi:10.1038/s41419-025-08365-7)
Supplement: Supplementary file 1 — Supplementary Figure [file 41419_2025_8365_MOESM1_ESM.docx]

**Fig. S1: Expression levels of PPP genes in luminal epithelial cells.**

**
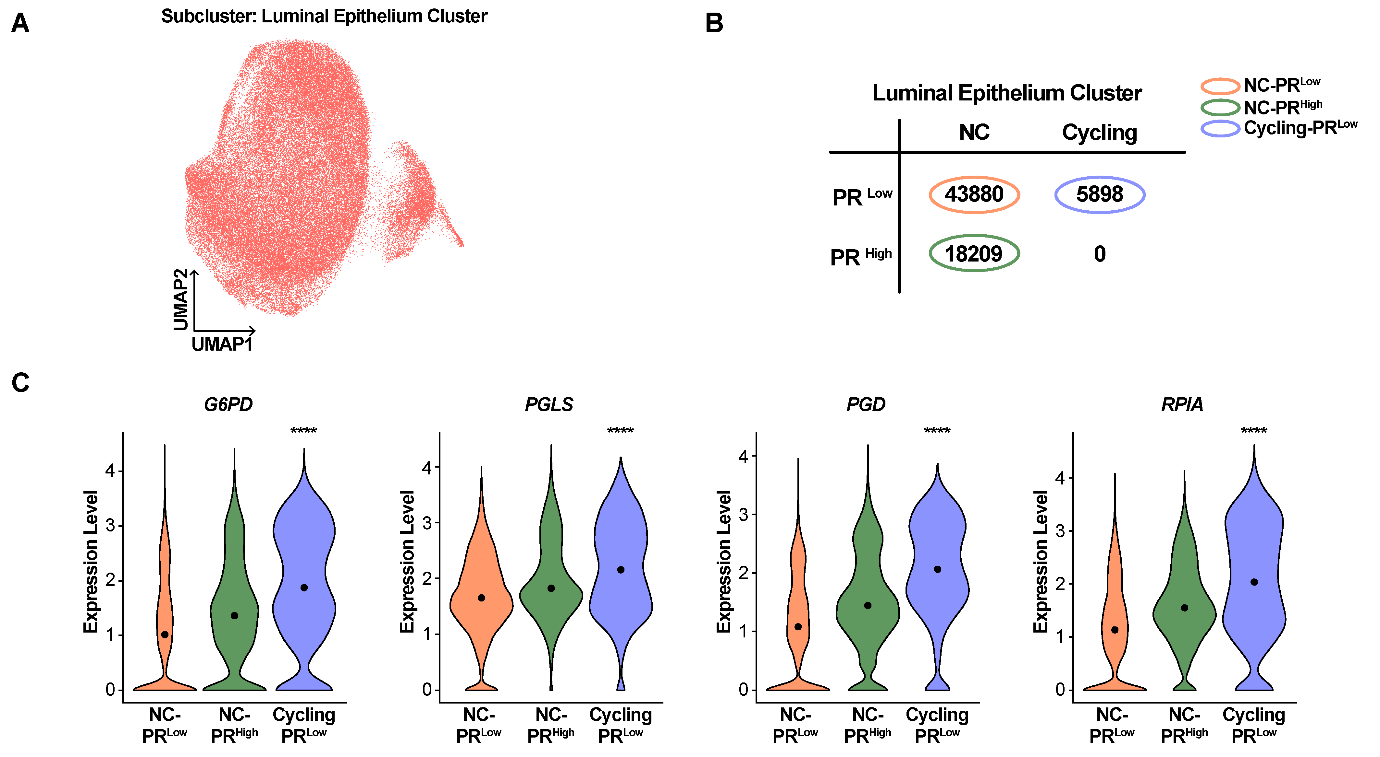
**

**A** UMAP plot of luminal epithelial cells, subsetted from the eight cell clusters. **B** Contingency table quantifying the number of cells within the NC and Cycling groups, further stratified by PR expression status. **C** Violin plots comparing expression levels of *G6PD*, *PGLS*, *PGD* and *RPIA* among the three groups. Statistical significance was assessed using the Wilcoxon rank-sum test, and *p-*values are shown for each comparison. * *p* < 0.05, ** *p* < 0.01, *** *p* < 0.001, **** *p* < 0.0001.

**Fig. S2: Analysis of the aneuploid luminal epithelial cells.**

**
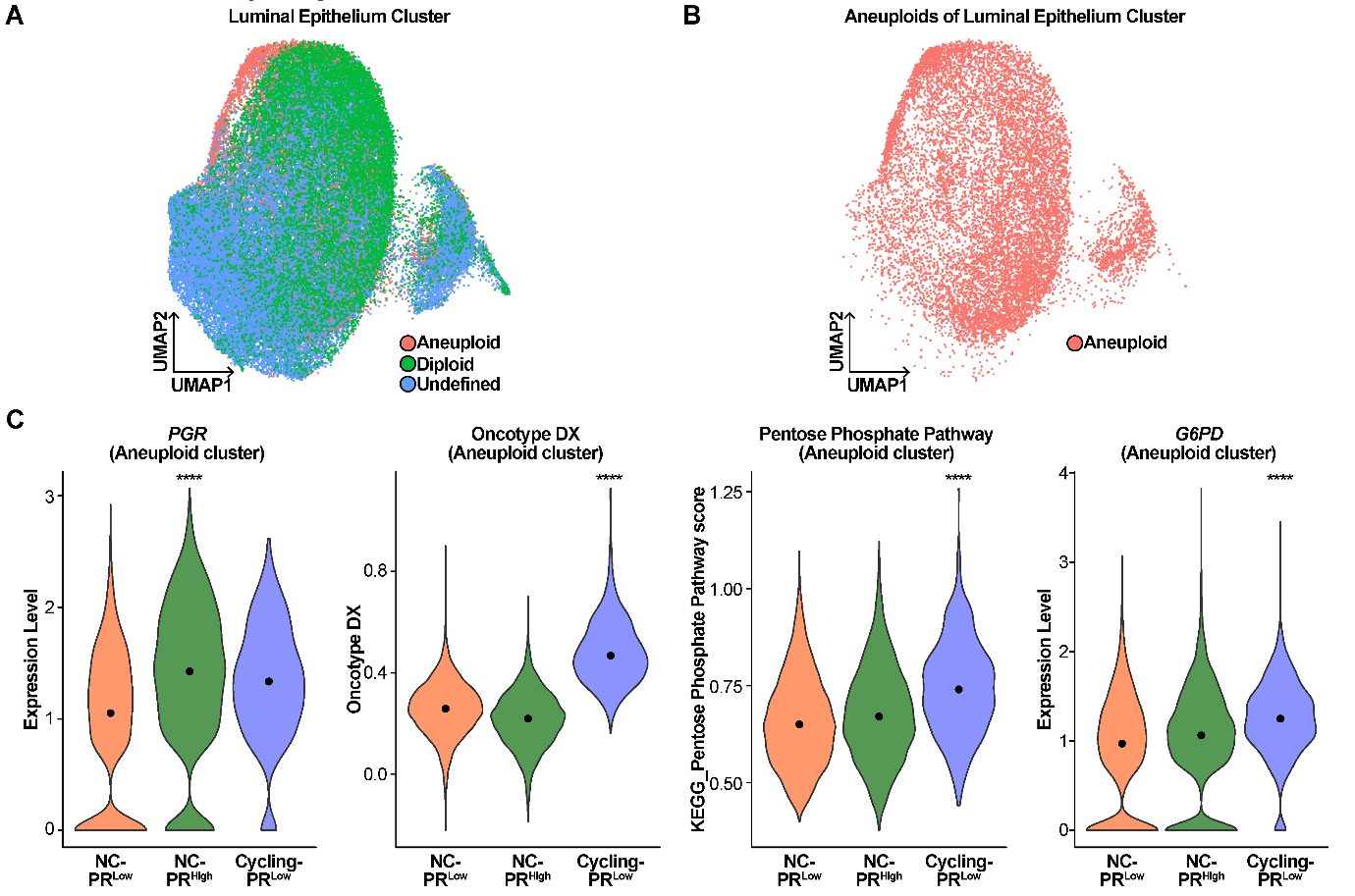
**

**A** UMAP plot of luminal epithelial cells colored by CNV status (Aneuploid, Diploid, Undefined). **B** UMAP visualization of the subsetted aneuploid cell population. **C** Violin plots comparing expression levels of PGR and G6PD, Oncotype DX (RS) score, and PPP score among the three subgroups within the aneuploid cells. Statistical significance was assessed using the Wilcoxon rank-sum test, and *p*-values are shown.

**Fig. S3: Refined analysis of transcriptionally divergent patient subgroups enhances the association between low PR status and PPP activation.**

**
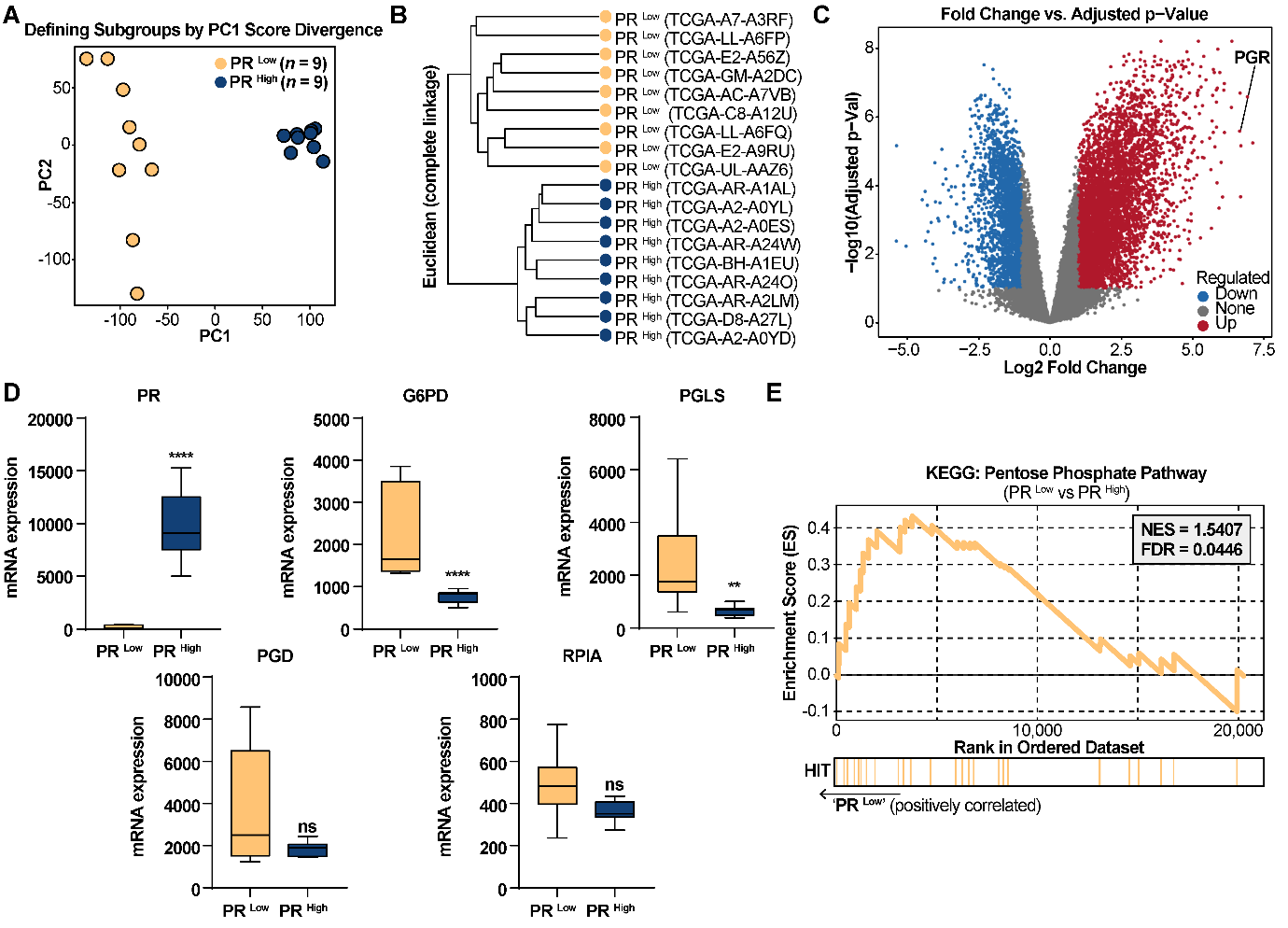
**

**A** PCA plot of the TCGA luminal cohort, illustrating the selection of the 'PR ^Low^ divergent' and 'PR ^High^ divergent' subgroups based on their divergent scores along the PC1 axis. **B** Hierarchical clustering demonstrating a clear segregation of the divergent subgroups by transcriptomic profile. **C** Volcano plot of DEGs between the PR ^Low^ divergent and PR ^High^ divergent subgroups, indicating a more pronounced transcriptomic divergence compared with the full cohort analysis. **D** Box plots comparing expression levels of *PGR* and PPP enzymes (*G6PD*, *PGLS*, *PGD*, *RPIA*) in the divergent subgroups. The upregulation of *G6PD* and *PGLS* is highly significant. **E** GSEA enrichment plot confirming significant enrichment of the PPP in the PR ^Low^ divergent group. For box plots, the line represents the mean value. Statistical significance was assessed using an unpaired two-tailed t-test. * *p* < 0.05, ** *p* < 0.01, *** *p* < 0.001, **** *p* < 0.0001.

**Fig. S4: The association between low PR expression and PPP gene activation is specific to the luminal subtype.**

**
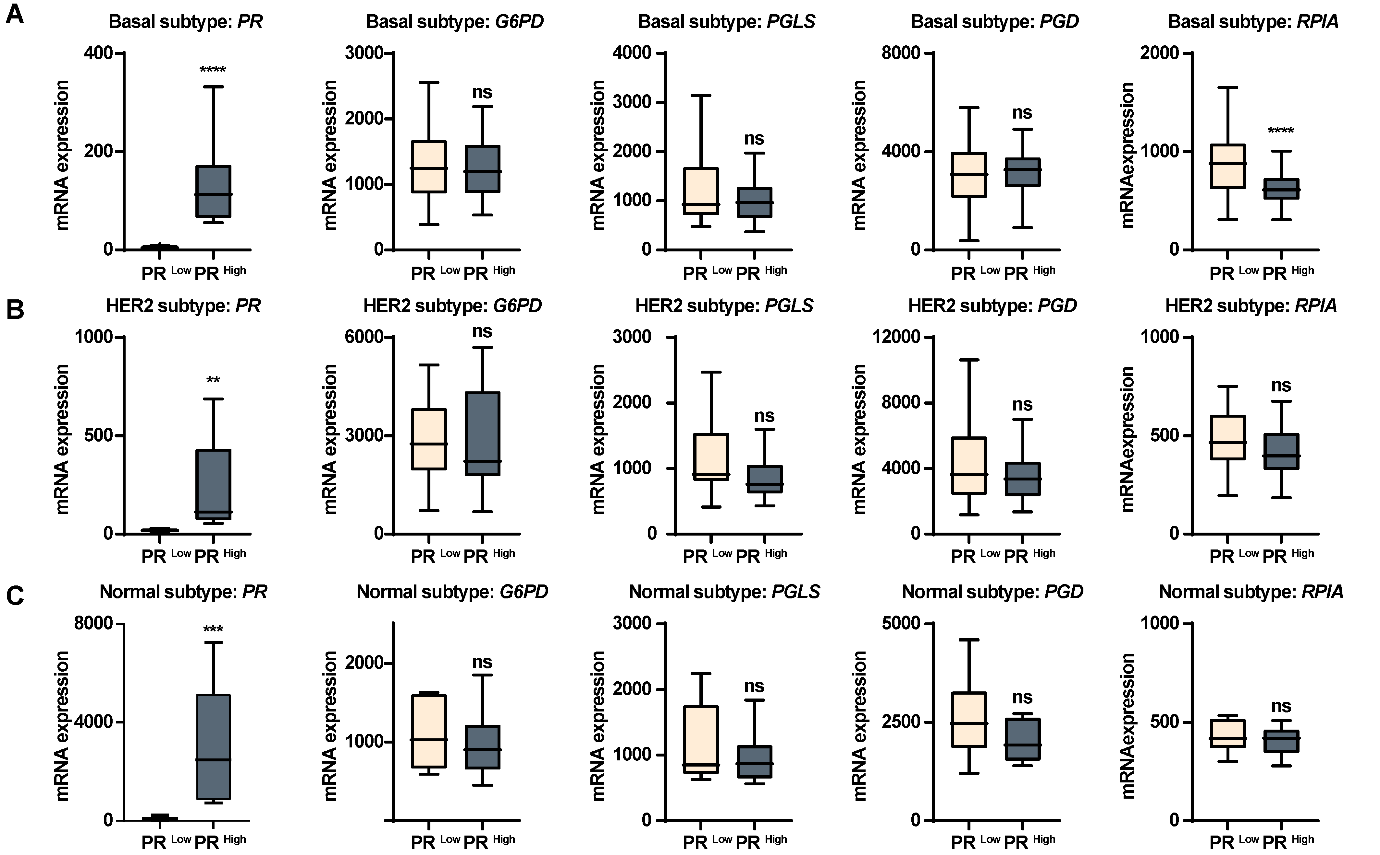
**

**A-C** Box plots comparing the expression of key PPP enzymes (*G6PD*, *PGLS*, *PGD*, and *RPIA*) between PR ^Low^ and PR ^High^ patient groups from the TCGA cohort within the **A** basal-like, **B** HER2-enriched, and **C** normal-like breast cancer subtypes from the TCGA cohort. Statistical significance was assessed using an unpaired two-tailed t-test. * *p* < 0.05, ** *p* < 0.01, *** *p* < 0.001, **** *p* < 0.0001.

**Fig. S5: Immunohistochemical scoring and classification of G6PD expression.**


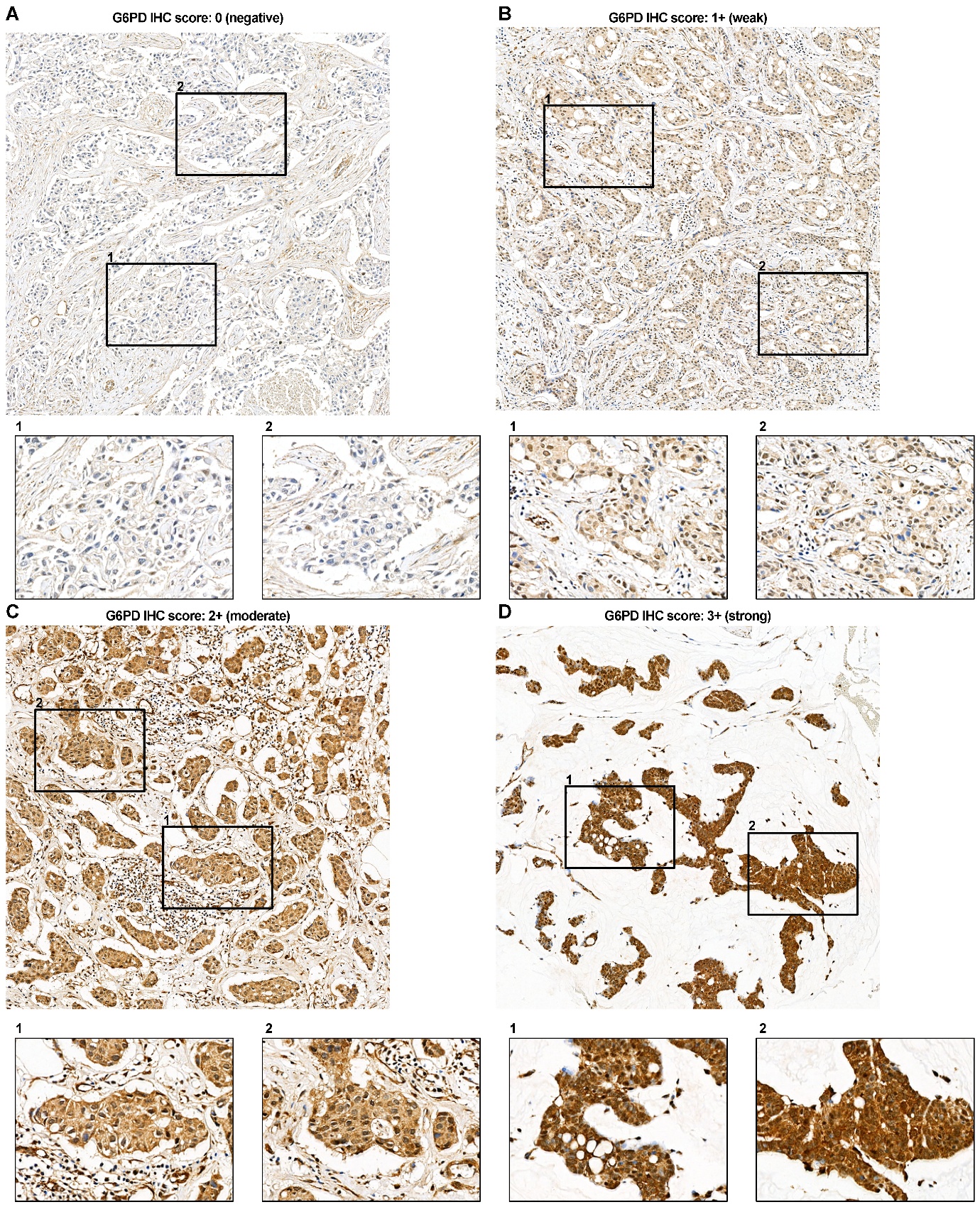


Representative IHC images showing the full scoring spectrum for G6PD protein expression in luminal-like breast cancer tissues from the Yonsei cohort. Panels correspond to staining intensity scores of **A** 0 (negative), **B** 1+ (weak), **C** 2+ (moderate), and **D** 3+ (strong). Patients with scores of 0 or 1+ were classified as G6PD ^Low^, while patients with scores of 2+ or 3+ were classified as G6PD ^High^. All images are shown at 400× magnification.

**Fig. S6: Prognostic significance of PR and G6PD expression in luminal-like breast cancer.**

**
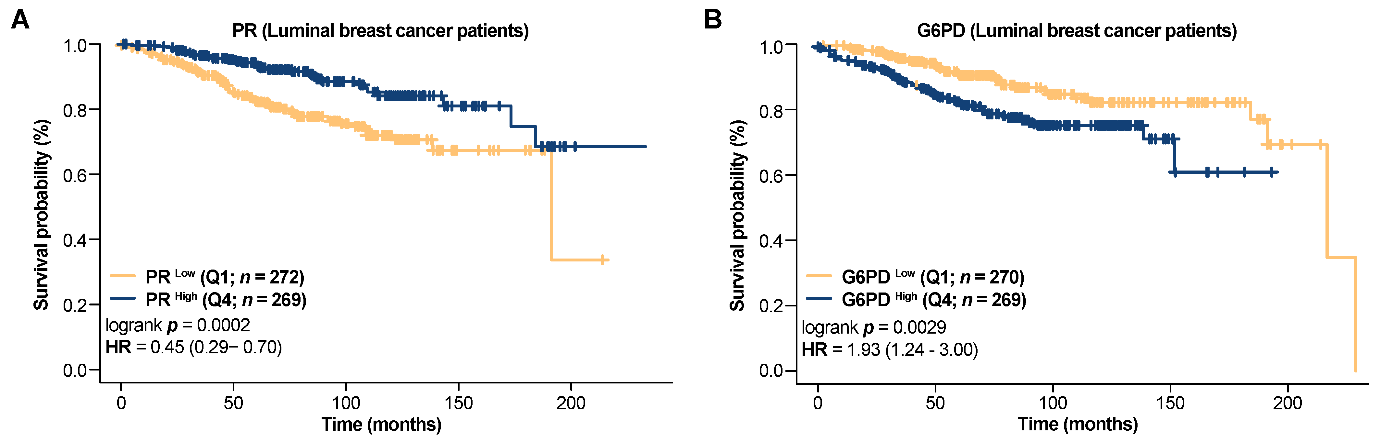
**

Kaplan–Meier survival curves for patients with luminal-like breast cancer analyzed using the KM-plotter web tool, which utilizes large-scale public datasets. **A** Comparison of overall survival between patients with high *PGR* expression (PR ^High^; top quartile) and low *PGR* expression (PR ^Low^; bottom quartile). **B** Comparison of overall survival between patients with high *G6PD* expression (G6PD ^High^; top quartile) and low G6PD expression (G6PD ^Low^; bottom quartile). Hazard ratios (HR) and log-rank *p*-values are shown for each comparison.

**Fig. S7: Individual replicate values from clonogenic formation assay.**


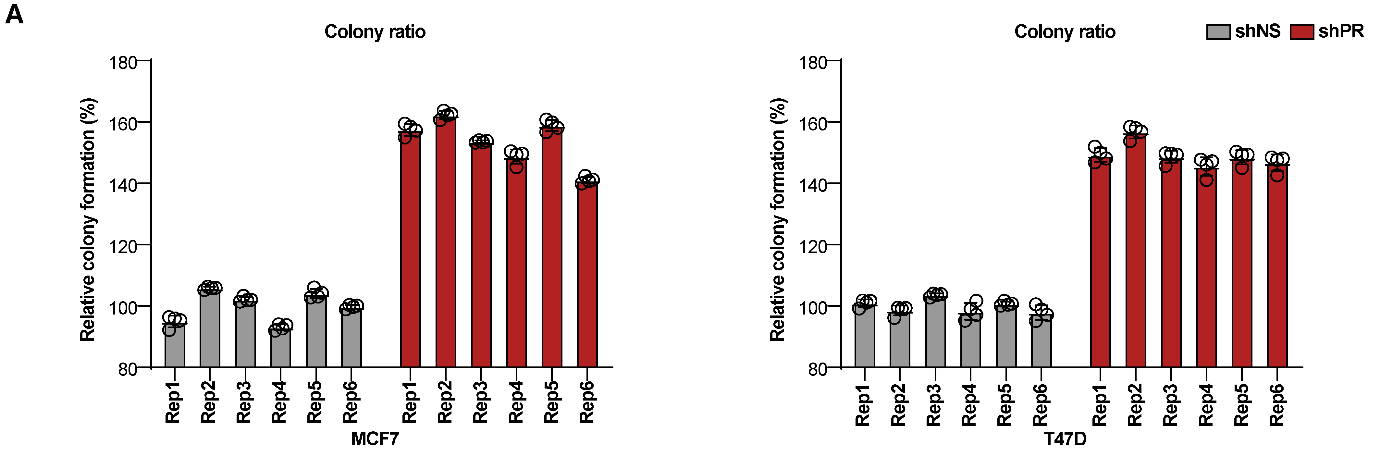


**A** Bar plots showing relative colony formation (%) from independent biological replicates in MCF7 and T47D cell lines. Colony formation for each replicate (shNS and shPR) is normalized to mean of the shNS group (set as 100%). These data illustrate experimental consistency and correspond to the summary graph (mean ± SD) in Fig. 4E.

**Fig. S8: Rescue experiment via PR re-expression in PR KD cells.**

**
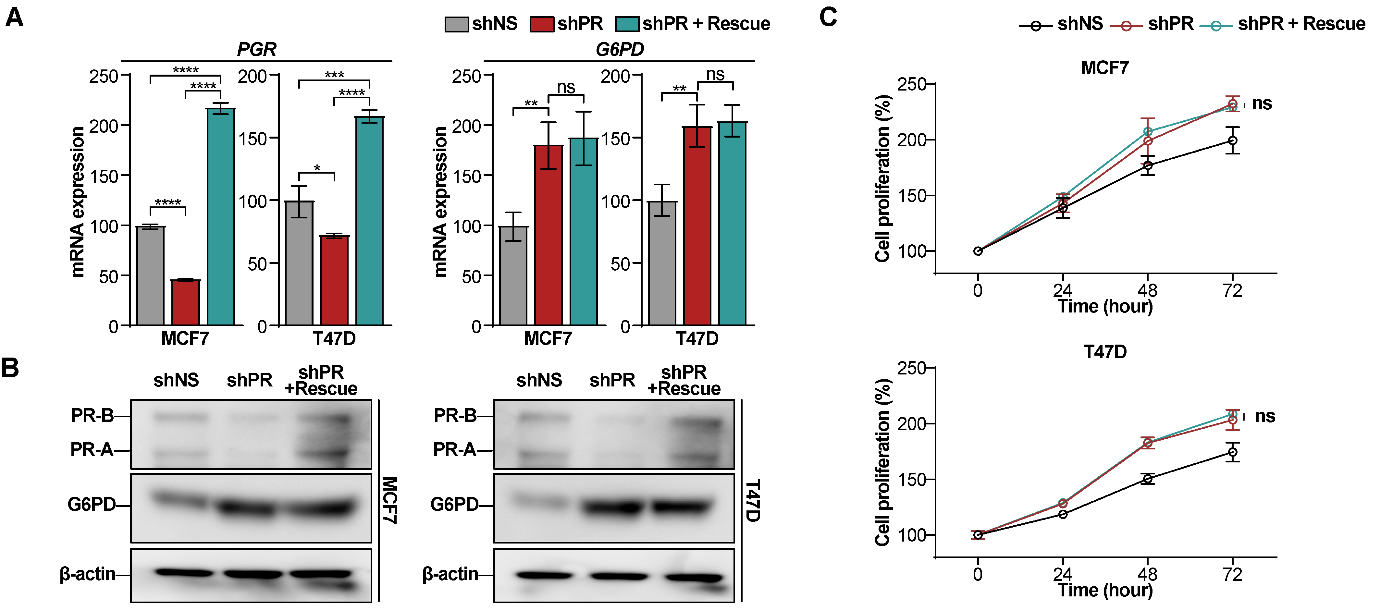
**

**A–B** Re-expression of PR in PR KD cells successfully restored PR expression levels but did not suppress G6PD upregulation at either mRNA or protein levels. **C** Proliferation assay revealed comparable growth rates between the PR rescue group and PR KD cells, suggesting that PR re-expression does not restore the proliferative phenotype. Data is presented as mean ± SD. Statistical significance was determined by unpaired two-tailed t-test. * *p* < 0.05, ** *p* < 0.01, *** *p* < 0.001, **** *p* < 0.0001.

**Fig. S9: Quality control and overview of bulk RNA-seq data.**


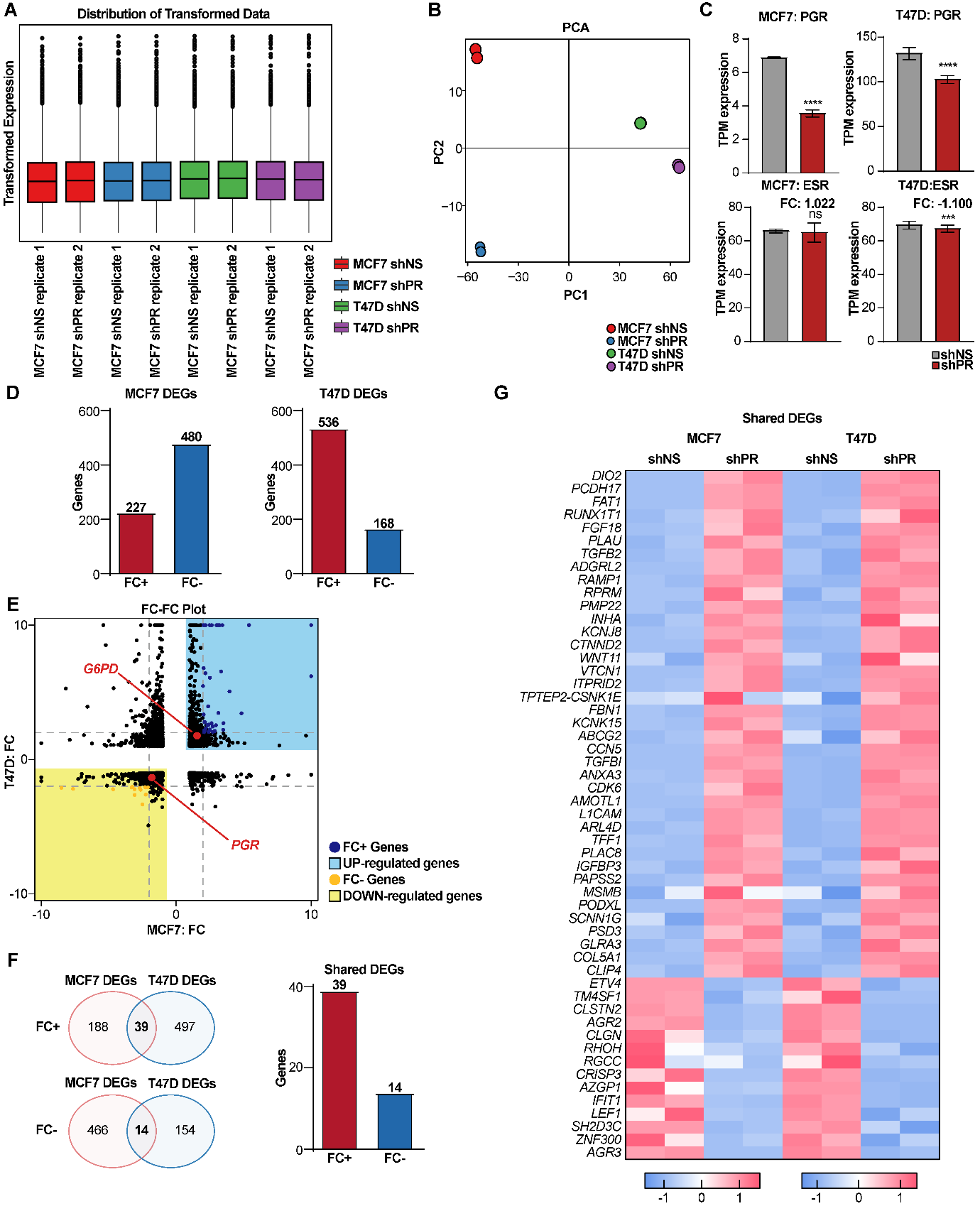


**A** Box plots showing the overall distribution of gene expression values across all samples, confirming comparable distributions after normalization. **B** PCA plot of transcriptomic data demonstrating clear separation between shNS and shPR. **C** Bar plots confirming efficient PR KD by showing *PGR* and *ESR1* expression at the transcript level in shPR compared with controls. **D** Bar plots summarizing the number of significantly upregulated and downregulated DEGs in each cell line. **E** Scatter plot comparing MCF7 fold-change and T47D fold-change, highlighting *PGR* and *G6PD*. **F** Venn diagrams (left) illustrating the overlap of upregulated (FC+) and downregulated (FC−) DEGs between MCF7 and T47D cells; a bar plot (right) summarizes the number of shared upregulated and downregulated DEGs. **G** Heatmap showing expression profiles of the 39 shared upregulated genes across MCF7 and T47D cells (shNS vs. shPR).

**Fig. S10: GSEA identified metabolic reprogramming in PR KD cells.**

**
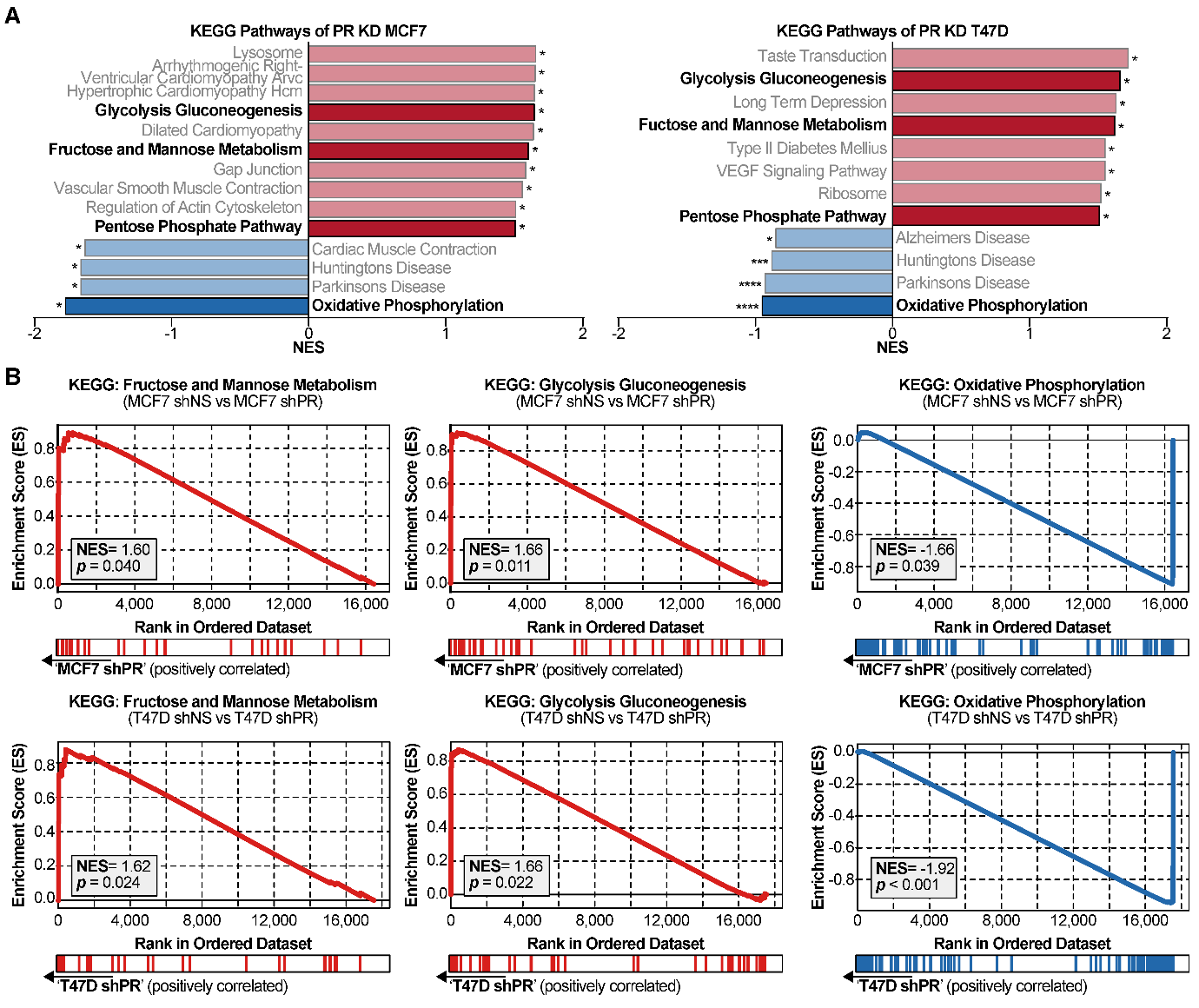
**

**A** Bar plot summarizing significantly enriched KEGG pathways (*p* < 0.05) in PR KD MCF7 and T47D cells identified by GSEA. This plot, filtered to show only pathways with significant *p*-values, demonstrates that glucose metabolism pathways, such as Glycolysis and the PPP, were significantly upregulated, while OXPHOS was significantly downregulated in both cell lines. **B** Detailed GSEA enrichment plots for key metabolic pathways.

**Fig. S11: Identification of commonly regulated transcription factors by PR KD.**

**
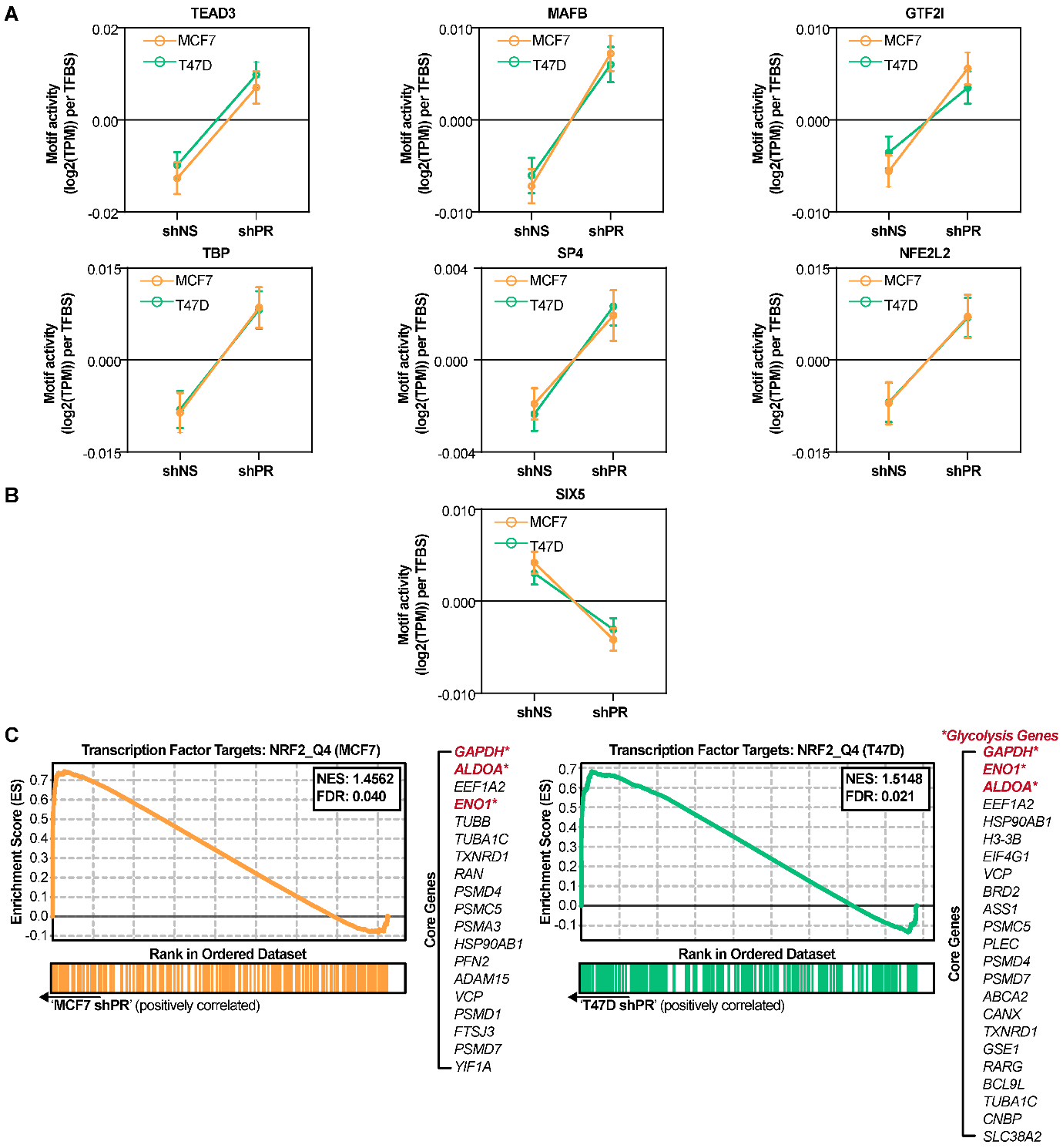
**

**A-B** Comparative plots of transcription factor motif activity (Z-score), predicted by ISMARA, in control versus PR KD cells. The plots highlight six commonly activated transcription factors (including *NFE2L2*/NRF2) and one commonly repressed factor across both MCF7 and T47D cell lines. **C** GSEA enrichment plots of the Transcription Factor Targets gene set show that NRF2 target genes are significantly upregulated in PR KD cells. Notably, several core NRF2 targets, such as *GAPDH*, *ALDOA*, and *ENO1*, are key components of glycolytic pathways, suggesting a functional link between NRF2 activation and metabolic reprogramming in both MCF7 and T47D cell lines.

**Fig. S12: NRF2 expression and functional binding motif analysis.**

**
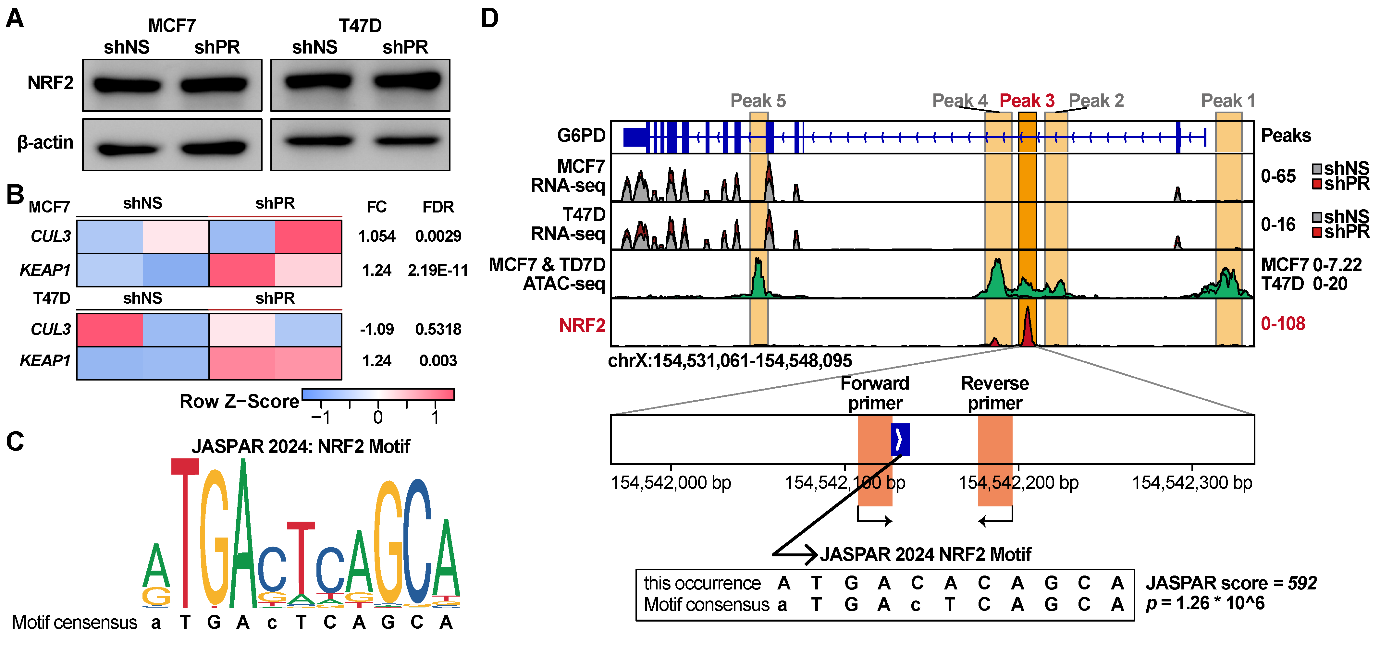
**

**A** Immunoblot analysis of total NRF2 protein levels in control and PR KD cells, showing that PR KD does not alter NRF2 protein expression. **B** Heatmap of *CUL3* and *KEAP1* expression, key NRF2 regulators, in MCF7 and T47D cells under shNS and shPR conditions, accompanied by fold-change and FDR values. **C** Canonical NRF2 binding motif sequence logo from the JASPAR 2024 database. **D** Genome browser view of the G6PD enhancer element (Peak 3), highlighting the NRF2 binding motif (*p* = 1.26E-06). Forward and reverse primer positions used for ChIP–qPCR are shown flanking the motif, indicating the amplified region.

**Fig. S13: Early oxidative response and G6PD induction following transient PR KD by siRNA.**

**
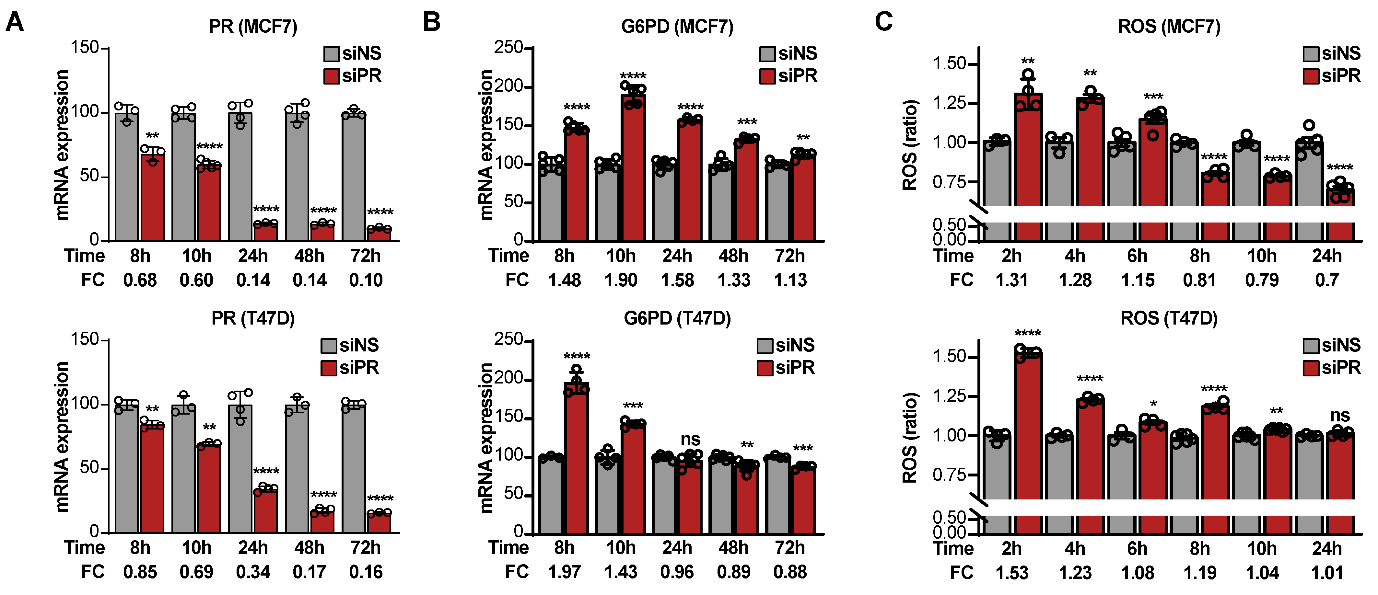
**

**A–B** Time-course analysis of PR and G6PD mRNA expression in MCF7 and T47D cells after transient PR silencing by siRNA at the indicated time points. **C** Intracellular ROS levels measured at the indicated time points after siPR transfection. A transient increase in ROS was observed during the early phase of PR KD, followed by induction of G6PD expression. The plot displays mean values ± SD. Statistical significance was determined using an unpaired two-tailed t-test. * *p* < 0.05, ** *p* < 0.01, *** *p* < 0.001, **** *p* < 0.0001.

**Fig. S14: NRF2 enhancer occupancy is unaffected by PR re-expression.**

**
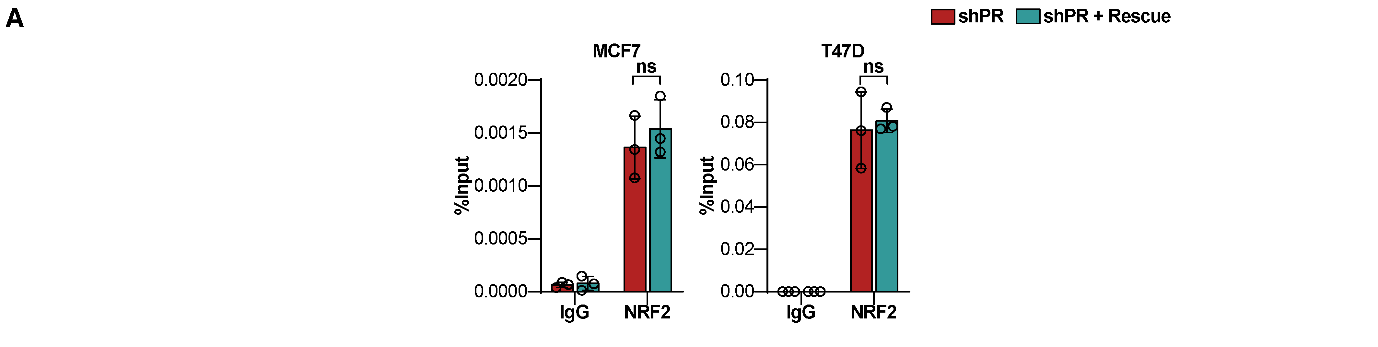
**

**A** ChIP–qPCR analysis showing sustained NRF2 binding at the G6PD enhancer (Peak 3) in PR KD cells following PR re-expression, indicating that NRF2 enhancer occupancy is not altered by PR rescue. The plot displays mean values ± SD. Statistical significance was determined using an unpaired two-tailed t-test. * *p* < 0.05, ** *p* < 0.01, *** *p* < 0.001, **** *p* < 0.0001.

**Fig. S15: Combination therapy of tamoxifen and G6PDi inhibiting aggressiveness of luminal breast cancer cells.**

**
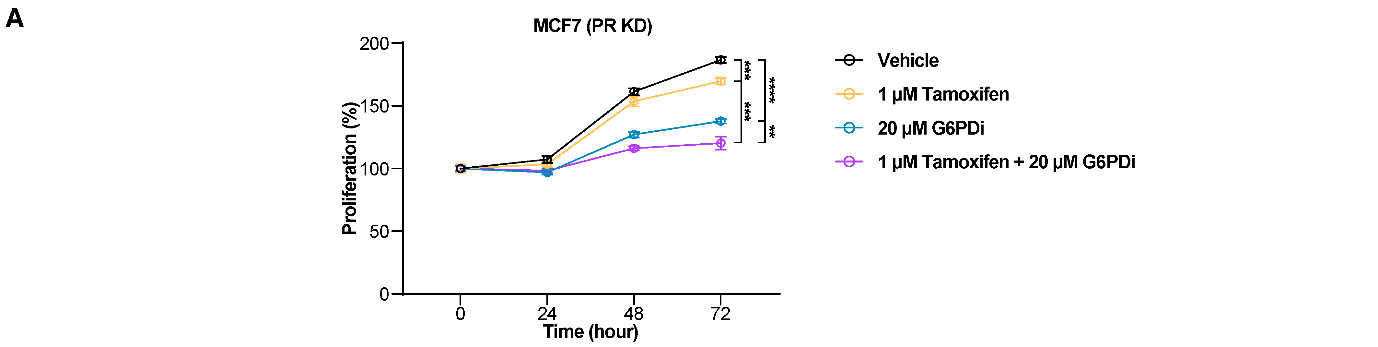
**

**A** Proliferation rate plot comparing vehicle, tamoxifen, G6PDi, and the combination of tamoxifen and G6PDi treatments in PR KD MCF7 cells. The proliferation rate was calculated as a relative value normalized to the 0-hour time point. The plot displays mean values ± SD. Statistical significance was determined using an unpaired two-tailed t-test. * *p* < 0.05, ** *p* < 0.01, *** *p* < 0.001, **** *p* < 0.0001.
