# Supplementary figures and images for: PR inhibition stimulates G6PD expression to enhance malignancy in luminal breast cancer
Source: Cell Death Dis. 2025 Dec 21;17(1):104. doi: 10.1038/s41419-025-08365-7 (PMC12847734; doi:10.1038/s41419-025-08365-7)

Fig. 4C

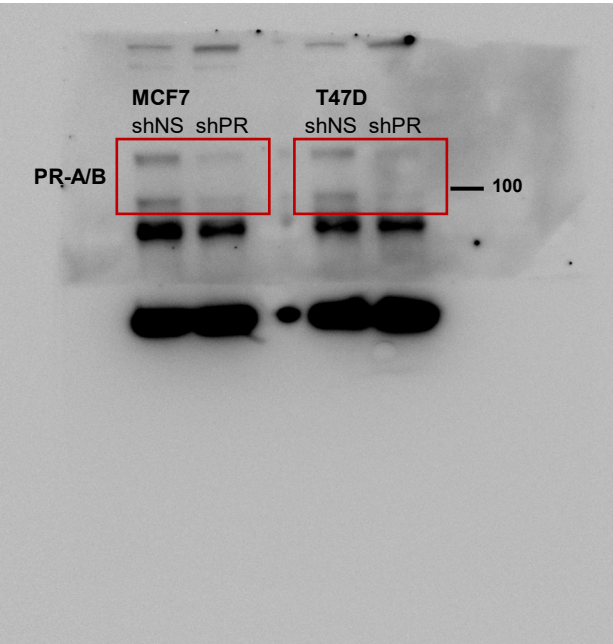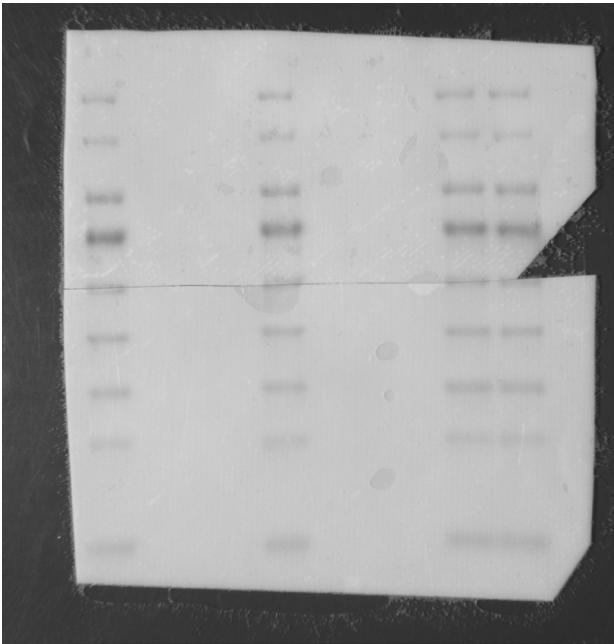

kDa

— 180  
— 130  
— 100  
— 70  
— 55  
— 40  
— 35  
— 25  
— 15

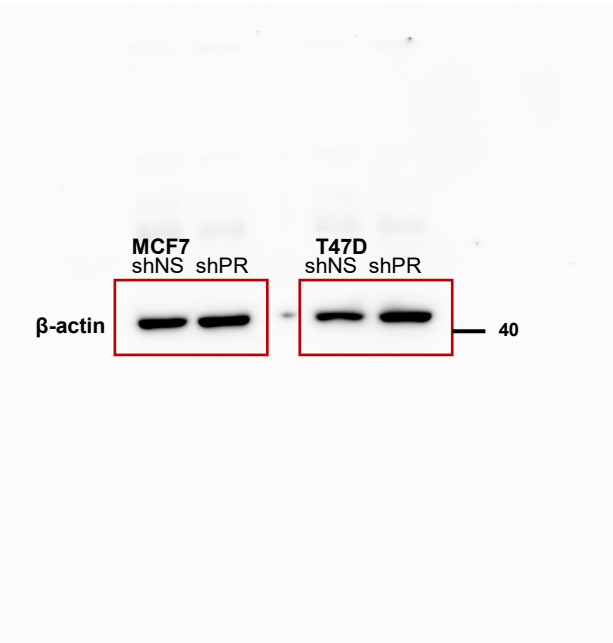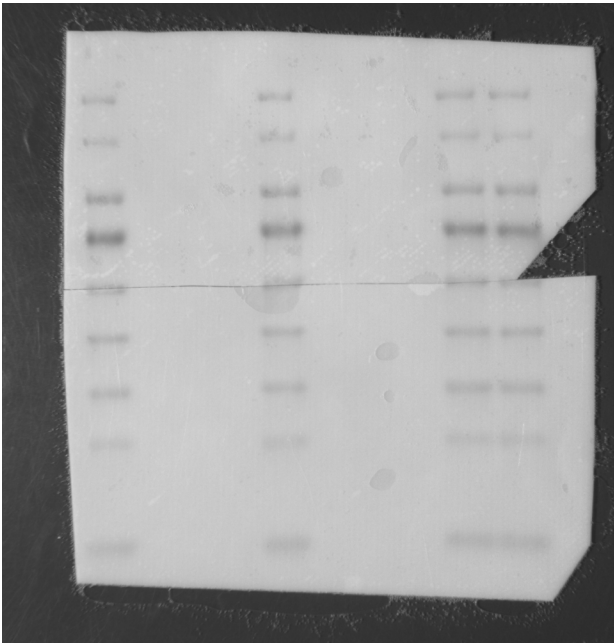

kDa

— 180  
— 130  
— 100  
— 70  
— 55  
— 40  
— 35  
— 25  
— 15

Fig. 6F

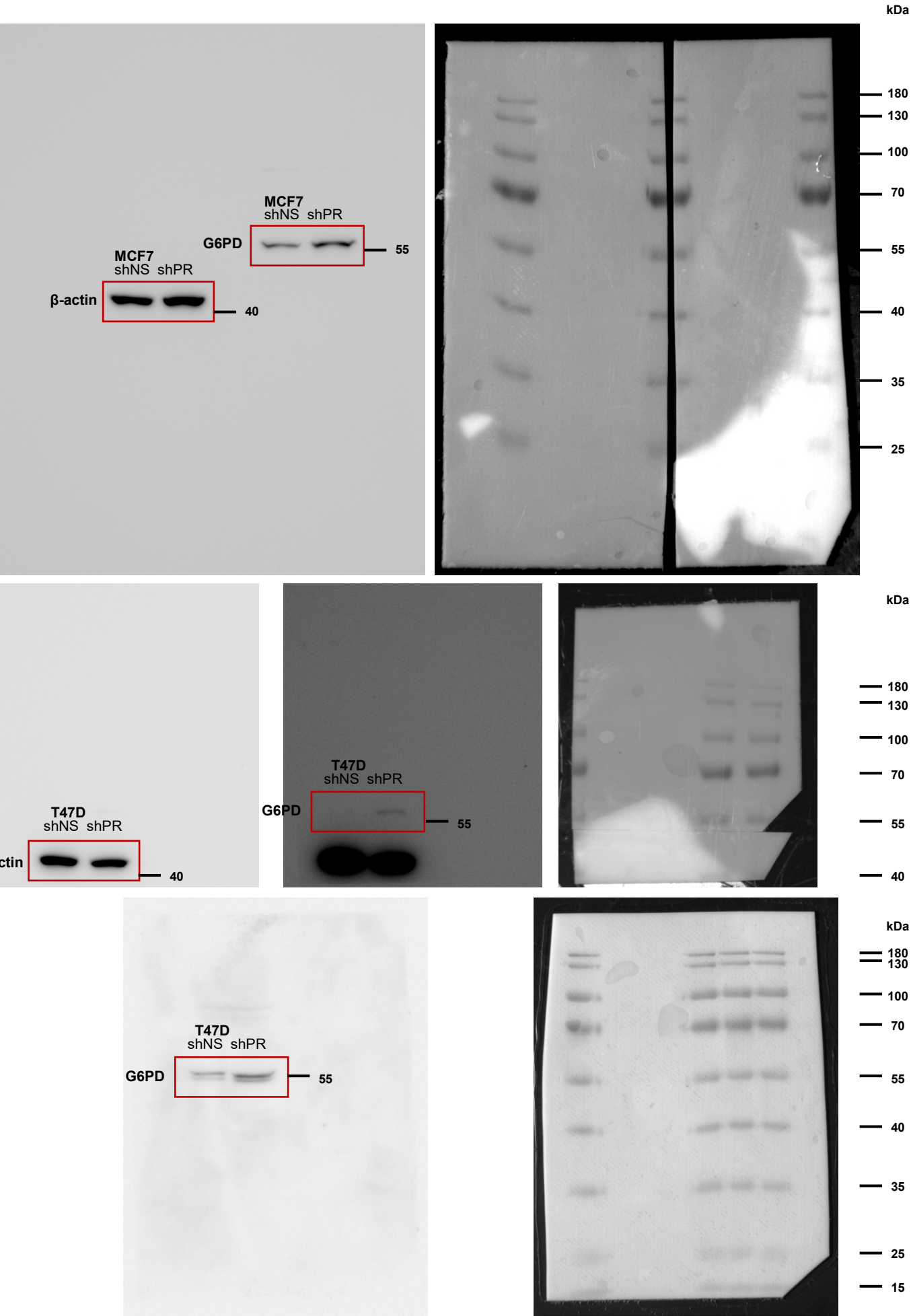

Fig. S8B

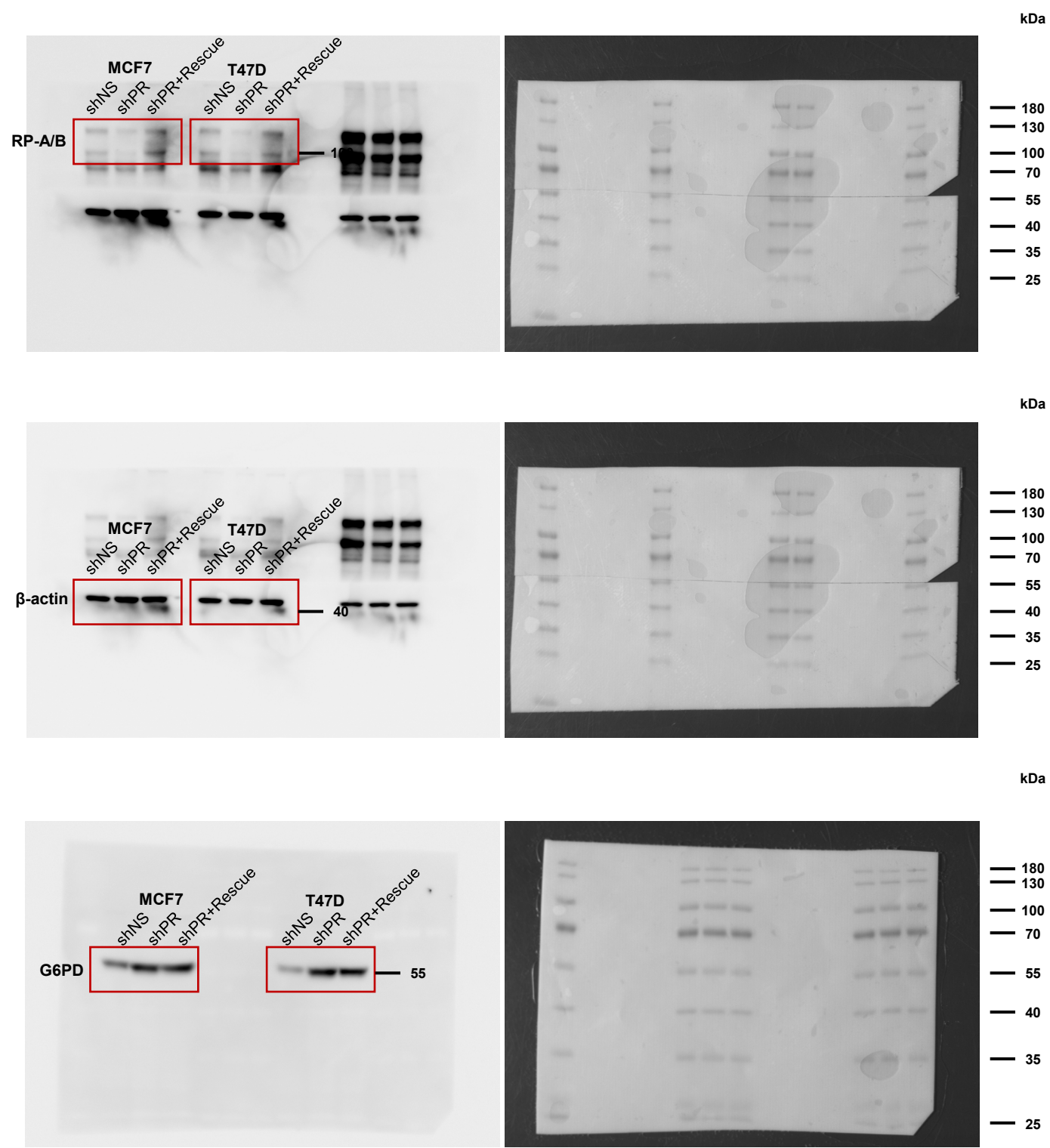

Fig. S12A

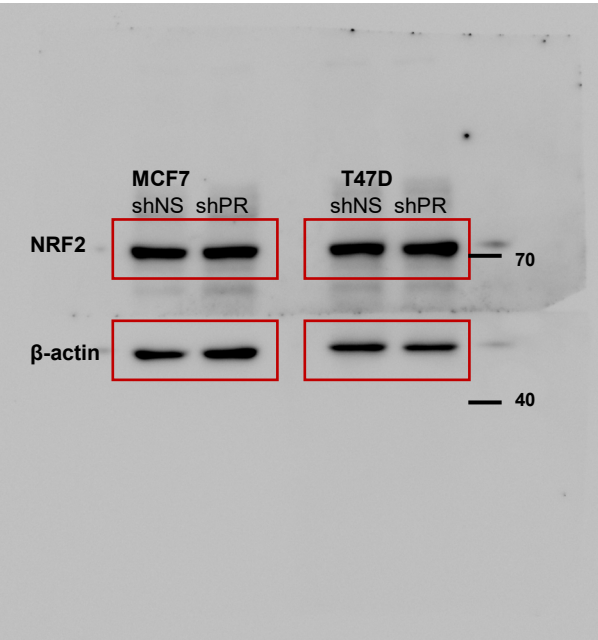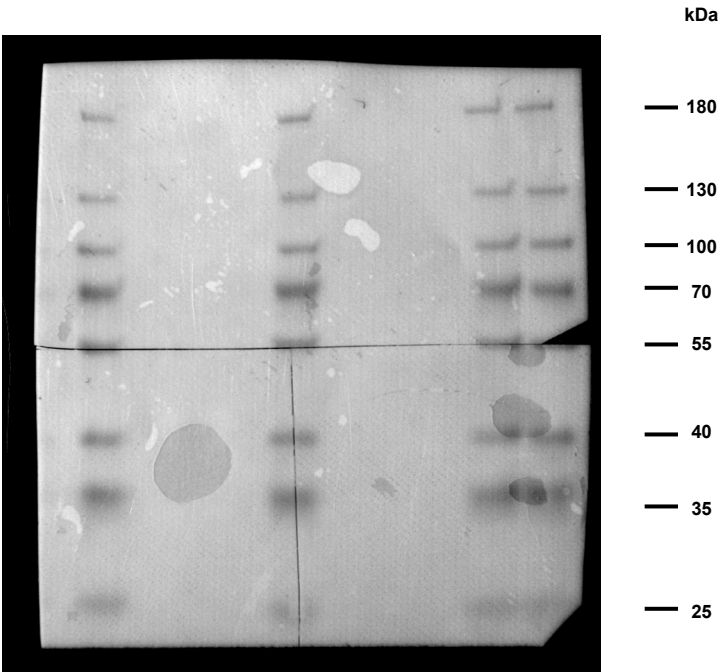

Supplement: Supplementary file 2 — Uncropped Western Blots [file 41419_2025_8365_MOESM2_ESM.pdf]
